# A Comparison of Methods for Modeling Multistate Cancer Progression Using Screening Data with Censoring after Intervention

**DOI:** 10.1177/0272989X261422681

**Published:** 2026-03-13

**Authors:** Eddymurphy U. Akwiwu, Veerle M. H. Coupé, Johannes Berkhof, Thomas Klausch

**Affiliations:** Amsterdam UMC, Vrije Universiteit Amsterdam, Department of Epidemiology and Data Science, Amsterdam Public Health, Amsterdam, The Netherlands; Amsterdam UMC, Vrije Universiteit Amsterdam, Department of Epidemiology and Data Science, Amsterdam Public Health, Amsterdam, The Netherlands; Amsterdam UMC, Vrije Universiteit Amsterdam, Department of Epidemiology and Data Science, Amsterdam Public Health, Amsterdam, The Netherlands; Amsterdam UMC, Vrije Universiteit Amsterdam, Department of Epidemiology and Data Science, Amsterdam Public Health, Amsterdam, The Netherlands

**Keywords:** cancer screening and surveillance, interval-censored data, multistate models, natural history, simulation study, survival analysis

## Abstract

**Background:**

Optimizing cancer screening and surveillance frequency requires accurate information on parameters such as sojourn time and cancer risk from premalignant lesions. These parameters can be estimated using multistate cancer models applied to screening or surveillance data. However, the performance of these models has not been thoroughly investigated in settings in which cancer precursors are treated upon detection, preventing progression to cancer. Our main goal is understanding the performance of available multistate methods in this challenging censoring setting.

**Methods:**

We assumed progression hazards between consecutive health states in a 3-state model (healthy [HE], cancer precursor, and cancer) to be either time independent or dependent on time since state entry and compared 6 methods implemented in R software packages with varying assumptions: time-independent hazards (msm), hazards dependent on time since state entry (msm with a phase-type model, cthmm, smms, BayesTSM), and hazards dependent on time since the start of the process (hmm). Risk estimates from each method were compared in simulations and illustrated using colorectal cancer surveillance data from 734 individuals, classified into 3 health states: HE, non-advanced adenoma (nAA), and advanced neoplasia (AN).

**Results:**

All methods performed well with time-independent hazards in the simulation study. With hazards dependent on time since state entry, only smms and BayesTSM provided unbiased risk estimates. In the application, only msm,hmm, and BayesTSM yielded converged solutions. The nAA risk estimates were similar between hmm and BayesTSM but differed for msm, while AN risk estimates varied across methods.

**Conclusions:**

Methods for multistate cancer models, specifically with unobservable precursor-to-cancer transition, are strongly affected by the time dependency of the hazard. With time-dependent hazards since state entry, BayesTSM provided robust estimates, in both the simulation and application.

**Highlights:**

- This study presents the first comprehensive comparison of available multistate modeling options for screening and surveillance data, focusing on the specific setting of a 3-state progressive model (healthy, cancer precursor, cancer) in which cancer precursors are treated upon detection so that the transition to cancer is prevented (censoring after intervention). Sample R code and simulated data demonstrating the compared methods, along with documentation (including installation instructions, manual, and/or worked examples) for the corresponding R software packages, are available at https://github.com/EddymurphyAkwiwu/MultiStateMethods.
- All methods provide unbiased risk estimates for transition times when the true progression hazards are time independent. With more realistic models in which progression hazards are dependent on time since state entry, only BayesTSM and smms yield unbiased risk estimates for transition times.
- In situations with weakly identifiable likelihoods, the smms package may suffer from numerical and optimization problems. The BayesTSM package overcomes these problems by applying regularized parameter estimation using weakly informative priors.
- Methods for multistate cancer models, more specifically with unobservable precursor-to-cancer transition, are strongly affected by the time dependency of the hazard. An inappropriate choice can lead to biased parameter estimates.

The primary goal of cancer screening and surveillance is the early detection of cancer and its precursor lesions, at a stage at which treatment is still possible. In cervical cancer screening, for example, women who test positive for human papillomavirus and have minor or more severe cellular abnormalities are usually referred for colposcopic examination, during which biopsies are taken for histological diagnosis. Those diagnosed with high-grade cervical intraepithelial neoplasia (CIN 2/3) undergo intervention (i.e., treatment) through invasive procedures, such as the loop electrosurgical excision procedure.^1^ Similarly, in breast cancer screening, women who receive a positive mammogram result are referred for further evaluation, which may include a breast biopsy. Those diagnosed with high-risk noninvasive conditions, such as ductal carcinoma in situ, are then referred for a comprehensive intervention plan that may include surgery, radiation, and endocrine therapy.^2^ In colorectal cancer (CRC) screening, individuals with a positive fecal immunochemical test are referred for colonoscopic evaluation, during which CRC precursors are removed through an endoscopic intervention procedure (called polypectomy) and biopsies are taken for histological testing. Individuals detected and treated for advanced or high-risk adenomas (defined as adenomas 1 cm, high-grade dysplasia or villous elements, or ≥3 adenomas) are further referred for surveillance with colonoscopy, as they are at increased risk of developing additional adenomas or CRC.^3^

Although the tests and follow-up procedures offered in the aforementioned screening programs have been shown to be effective in reducing cancer incidence and cancer-related mortality, they are expensive, resource intensive, and can cause harm. For example, some involve invasive procedures that carry risks of complications, such as bleeding, infection, radiation exposure, or colon perforation.^4–10^ Consequently, there is a need to optimize cancer screening and surveillance frequency. To do so, accurate information on parameters such as sojourn time (i.e., the time spent in cancer prestates before transitioning to cancer) and cancer risk from premalignant lesions is essential.

One potential approach to estimating these parameters is by modeling the natural history of cancer using data from screening or surveillance programs. A common approach for this purpose is multistate models, such as progressive 3-state survival models applied to screening or surveillance data.^11–16^ These models typically include health states such as healthy (state 1), cancer precursor (state 2), and cancer (state 3), as depicted in Figure 1. We denote the progression times between consecutive states as x and t . States 1 and 2 are transient, while state 3 is absorbing and can be entered only from state 2. However, only few multistate methods (see the “Methods” section) and their associated software packages are known to practitioners. Moreover, the performance of methods for modeling screening or surveillance data is generally not well understood.

**Figure 1:**
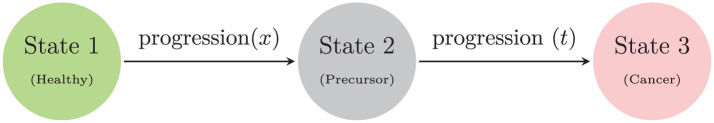
A progressive 3-state cancer model showing the underlying process. x and t represent the times from state 1 to 2 and from state 2 to 3, respectively. Hence, the time from state 1 to 3 (cancer) is x+t .

In this article, we present the first comprehensive comparison of available multistate modeling options for screening and surveillance data focusing on the specific setting in which the cancer precursor state is treated by intervention (e.g., surgical removal), which we refer to as screening data with censoring after intervention. Previous studies have evaluated multistate models in different settings.^17–19^ Specifically, if the cancer precursor state of diseases such as cervical, breast, or CRC is found during screening or surveillance, it is, typically, treated (i.e., removed), so that the transition to cancer is not observed (i.e., from state 2 to 3 in Figure 1). For example, in CRC screening and surveillance, advanced adenomas (AAs) are regarded as the clinically relevant precursors of CRC. Hence, after finding an AA, waiting to observe the growth of AA into CRC would be impossible, as AAs must be treated (i.e., removed) upon detection to prevent transitioning to CRC.^15,20^ An important complication that arises when working with screening or surveillance data is, furthermore, that the disease status is observed at only discrete time points and hence is subject to interval censoring.^15^ Consequently, the data that are typically obtained from screening or surveillance programs with interventions^21–24^ have a specific structure that, as we will demonstrate, can cause bias and other estimation problems in multistate models.

## Methods

### Model Assumptions and Data Structure

**Figure 2:**
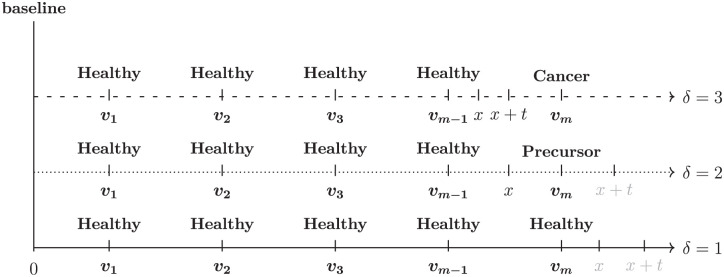
Observation process in cancer screening or surveillance programs involving an intervention, giving rise to the data structure considered in the present study. At baseline, everyone is assumed to be healthy, with no prevalent cases of cancer precursor lesions or cancer. The 3 timelines represent the 3 typical situations that may occur during screening or surveillance, with test moments $\left(v_{1},v_{2},\cdots \;,v_{m-1},v_{m}\right)$ leading to the 3 possible censoring states: healthy, cancer precursor, or cancer ( δ∈{1,2,3} ), respectively.

Throughout this study, we made several assumptions consistent with our previously published studies.^14–16^ First, we assumed noninformative censoring,^25^ meaning that the progression times (x,t) in Figure 1 are independent of the screening or surveillance visit times, denoted by v , at which screening tests are done (see Figure 2). This assumption holds either unconditionally or conditionally on the individual’s characteristics or the underlying disease process. Second, we assumed that the test determining an individual’s disease status during screening or surveillance is perfect and that everyone starts in the healthy state.

The data structure (see Supplement 1 for an example) arising from the observation process in cancer screening or surveillance programs in which disease progression, if detected, is interrupted by an intervention can be described as follows. Let $v=\left(v_{1},v_{2},\cdots \;,v_{m-1},v_{m}\right)$ represent the visit times at which screening or surveillance tests are performed, as illustrated in Figure 2. There are 3 typical situations that may arise during these visit times, leading to the classification of individuals into the censoring health states depicted in Figure 1. We denote these health states (healthy, cancer precursor, cancer) as δ∈{1,2,3} .

Individuals with δ=1 (bottom panel) do not develop a cancer precursor or cancer before the last screening or surveillance visit $v_{m}$ . Consequently, an event (i.e., a cancer precursor or cancer) can happen only after the last visit $v_{m}$ . These individuals are said to be right censored at $v_{m}$ .

Individuals with δ=2 (middle panel) are detected with a cancer precursor at $v_{m}$ , with the potential to progress to cancer if left untreated (i.e., not removed). We are interested in only the time of first detection of an event (i.e., a cancer precursor or cancer) in individuals during screening or surveillance from baseline because upon detection, these individuals undergo an intervention (i.e., the cancer precursor is treated and removed) and are censored, meaning that any subsequent visits are disregarded, and the progression to cancer is not observed. This implies that the cancer precursor must have developed within the interval $\left(v_{m-1},v_{m}\right]$ , resulting in interval censoring.

Individuals with δ=3 (top panel) are detected with cancer at $v_{m}$ . They are treated and immediately censored, with no further follow-up. This implies that both the transition from the healthy state to the cancer precursor state and then to cancer must have occurred within the same interval $\left(v_{m-1},v_{m}\right]$ , resulting in interval censoring as well.

### Methods for Multistate Models

In this section, we present statistical methods that we are aware of for modeling the progression of chronic diseases using screening data of the type described in the “Model Assumptions and Data Structure” section.^11–14,17,18,26–34^ These methods differ broadly in their assumptions regarding the form of the hazard functions that describe the rates of transitioning between health states and the observation process (i.e., data-censoring structure).

With respect to the form of the hazard functions, these methods can be classified as time-homogeneous Markov, time-inhomogeneous Markov, and semi-Markov models. In time-homogeneous Markov models, the sojourn time follows an exponential distribution in which the hazard rates are time-independent.^11–13,26,28^ In a time-inhomogeneous Markov model,^17,31,34^ the hazard rate is influenced by age (or, more generally, by the start of the process), and in a semi-Markov model,^14,18,29,30,33^ the hazard rate is influenced by the time since onset in a transient state.

In terms of the observation process, some methods rely on current status data,^11–13,27,32,33^ in which the health state of individuals is observed at only a single examination, making it impossible to observe the disease process over time. However, in most cancer screening and surveillance studies, individuals are repeatedly examined over time, which we denoted by v in Figure 2. This leads to interval-censored data as described above. Some methods^14,17,18,28–31^ have been developed to handle such type of data.

While the aforementioned literature offers a wide range of statistical methods for fitting multistate models, this study focuses on methods specifically applicable to progressive 3-state models (Figure 1) with and without covariates. In addition, we selected methods only capable of handling interval-censored data and for which implementation software code is publicly available. Based on the inclusion criteria, we selected the following methods for our study: msm,^28^msm with a phase-type model,^30^cthmm,^18^smms,^29^BayesTSM,^14^ and hmm^17^; see the “Overview of Compared Methods”. Note that, with the exception of a method developed in our previous study,^14^ these methods were included because they are all, at least in theory, presumably capable of modeling the specific type of data considered in this study, namely, cancer screening data with censoring after intervention.

### Overview of Compared Methods

This section provides a summary of the selected methods, outlining their underlying model assumptions, computational algorithms, ease of obtaining parameters of interest, and available documentation, with minimal mathematical detail. For more details, we refer the reader to the corresponding papers. In addition, sample R code for each method and documentation for the corresponding R software packages are available at https://github.com/EddymurphyAkwiwu/MultiStateMethods.

Although the parameters of interest that can be estimated differ for some methods (see Table 1), our primary emphasis is the cumulative incidence function (CIF), which is the cumulative probability of an event (i.e., cancer precursor or cancer) occurring at or before a specific time point. We will present both the marginal CIF, which is the CIF averaged over covariates in the population,^35^ and the conditional CIF, which is the CIF for a particular covariate profile.

**Table 1:** Summary of Selected Methods with R Software Packages for Implementing Multistate Models: Parameters of Interest and Model Assumptions

	Methods					
Parameter of Interest	msm	msm-phase	cthmm	hmm	smms	BayesTSM
Marginal CIF^a^	✓	✓	✓	✓	✓	✓
Credible or confidence interval for marginal CIF^b^	✓	✓	✓	✓	✓	✓
Conditional CIF^c^	✓	✓	✓	✓	✓	✓
Credible or confidence interval for conditional CIF^d^	✓	✓	✓	✓	✓	✓
Hazard ratio^e^	✓	✓	✓	✓	✓	✓
State occupancy probability^f^	✓	✓	**✗**	**✗**	✓	**✗**
Transition probability^g^	✓	✓	✓	✓	✓	**✗**
Statistics for sojourn time^h^	✓	✓	✓	**✗**	✓	✓
**Model assumption**						
Time-homogenous Markov model	✓	✓	✓	✓	✓	✓
Time-inhomogenous Markov model^i^	✓	**✗**	**✗**	✓	**✗**	**✗**
Semi-Markov model by parametric distribution	**✗**	**✗**	**✗**	**✗**	✓	✓
Semi-Markov by phase-type model^j^	**✗**	✓	✓	**✗**	**✗**	**✗**
Accelerated failure time model	**✗**	**✗**	**✗**	**✗**	✗	✓
Proportional hazards model	✓	✓	✓	✓	✓	✓
Informative censoring	**✗**	**✗**	✓	**✗**	**✗**	**✗**

CIF, cumulative incidence function; msm, standard multistate model; msm-phase, msm with phase type; cthmm, continuous-time hidden Markov models; hmm, hidden Markov model; smms, semi-Markov multistate models; BayesTSM, Bayesian 3-state model.

a For msm, CIF can be estimated using either the ppass.msm(), qmatrix.msm(), or pmatrix.msm() function; see Jackson^28,36^ for definitions. For msm-phase, this parameter can be estimated using the qmatrix.msm() function. See sample R code in the GitHub repository accompanying this article for demonstration. For smms, CIF can be computed directly using in-built R functions, such as pexp() and pweibull().

b For both msm and cthmm, this parameter can be estimated from the package only for models with no covariates. However, for models with covariates, nonparametric bootstrapping can be used. Estimating this parameter is not supported in either msm-phase or smms. However, nonparametric bootstrapping can be used for models both with and without covariates. For both hmm and BayesTSM, this parameter can be estimated directly from the package, regardless of whether the model includes covariates.

c For cthmm, conditional CIF estimates are attainable only for observed covariate values in the population. Predictions beyond the observed covariate are not supported.

d For both msm-phase and smms, nonparametric bootstrapping can be used, regardless of whether the model includes covariates. For other methods, this parameter can be estimated directly from the package, regardless of whether the model includes covariates.

e For BayesTSM, this requires transforming the regression estimates from an exponential or a Weibull accelerated failure time model to those of a proportional hazards model; see Collett^37(p237)^ for the approach.

f This has been referred to as *prevalence* in parts of the literature.^28,38^

g For definition, see Jackson,^28^ Aastveit et al.,^29^ and Jackson.^36^

h This includes at least 1 of the following: mean, median, or restricted mean estimates of the sojourn time. Note that sojourn time has been referred to as *waiting time* in parts of the literature.^39^

i In msm, this can be implemented using the pci argument in the main function msm().

j For msm-phase, phase-type models are used directly as sojourn distributions, while for *cthmm*, they are used to approximate generic distributions with positive support.

#### MSM

Kalbfleisch and Lawless^40^ proposed a maximum likelihood (ML) method for fitting multistate time-homogeneous Markov models. This method is implemented in the R package msm, developed by Jackson.^28^ The ML estimation is performed using the R optimization function optim. The package can also fit time-inhomogeneous Markov models by using the pci argument in the main function msm(), which assumes piecewise-constant hazards. It includes functions to compute various parameters of interest based on the estimated transition rate matrix, such as transition probabilities, which can be used to derive the marginal and conditional CIFs. The package includes a comprehensive reference manual, a vignette with worked examples, and an online course^36^ to guide users in implementing the package.

#### MSM-Phase

Titman and Sharples^30^ proposed an ML method for multistate models that assume semi-Markov processes, using Coxian phase-type distributions directly as the sojourn distributions. This method is implemented in the msm package via the phase.states() argument, where the user specifies a 2-phase distribution for the transient states. We refer to this implementation as msm-phase. As with the “standard”msm model, estimates of marginal and conditional CIFs can be indirectly computed using the estimated transition rate matrix. While the msm package includes an extensive documentation, there are limited resources available to guide users in implementing msm-phase.^36^

#### CTHMM

Similar to the approach by Titman and Sharples,^30^ Lange and Minin^18^ proposed an ML method for fitting semi-Markov multistate models that uses Coxian phase-type distributions, but only as an approximation. The method is implemented in the R package cthmm, which supports an arbitrary number of phases. The package employs an expectation–maximization algorithm to estimate the transition rates and includes a function to directly compute the conditional CIFs, from which the marginal CIFs can be readily derived. However, conditional CIF estimates are feasible for only observed covariate values in the population, with no support for predictions outside these values. While the package includes a reference manual, it may not provide sufficient guidance for implementing the method. Users may find it helpful to consult the example code from a related study^41^ or our sample R code to better understand the examples provided in the manual.

#### SMMS

A recent ML method for fitting semi-Markov multistate models was proposed by Aastveit et al.,^29^ available as R package smms. It allows parametric models to be specified for transition times in the underlying semi-Markov multistate model. The package uses the nlminb function in R to obtain ML estimates of the model parameters. While smms provides functions for computing various parameters of interest, it lacks a direct function for computing CIFs, which can be derived using built-in R distribution functions (e.g., pweibull()). Estimates of the marginal and conditional CIFs can be obtained afterwards. In addition, the package includes a vignette with worked examples to guide users in implementing the method.

#### BayesTSM

A Bayesian method for fitting progressive 3-state, semi-Markov models is our previously published model,^14^ implemented in R package BayesTSM. This model assumes the structure in Figure 1 with accelerated failure time (AFT) models for (x,t) . Similar to smms, parametric models can be specified for the (x,t) . The method is restricted to the data-censoring setting considered in this article (“Model Assumptions and Data Structure”). Parameter estimation is performed using a Metropolis-within-Gibbs Markov Chain Monte Carlo (MCMC) sampling algorithm and uses a multivariate normal proposal distribution. The method uses weakly informative priors for the model parameters to enhance the robustness of MCMC parameter estimation through regularization.^42^ The package provides functions to directly compute the marginal and conditional CIFs using MCMC samples of the model parameter estimates. It also includes well-annotated example code to guide users in implementing the method.

#### HMM

Williams et al.^17^ proposed a Bayesian method for multistate hidden Markov models. The method assumes a time-inhomogeneous Markov process, in which the transition rates are modeled as functions of an individual’s age at the time of transition, ensuring that the progression hazards change only when an individual’s integer age changes. A similar age-dependent model can also be fitted in the msm package (see Jackson^36^). Implemented in R software, the model parameters are estimated using the Metropolis-within-Gibbs sampling algorithm. This method employs a multivariate normal proposal distribution with multivariate normal priors for the model parameters. A custom set of R functions provided by the authors for implementing the method contains functions to compute the marginal and conditional CIFs using the MCMC samples containing the model parameter estimates. However, no reference manual or user guide is available; only the code required to reproduce the results of the original study is publicly available. Throughout this study, we refer to this implementation as hmm, following the terminology used by the authors.

Table 1 provides an overview of the methods discussed in this section, highlighting parameters of interest that can be estimated as well as the main model assumptions associated with each method. All methods can estimate the influence of risk factors measured by hazard ratios (HRs) in the development of an event (i.e., cancer precursor lesions or cancer) by assuming a proportional hazards model.

## Performance of Compared Methods

Previous studies^14,15,19,29^ have shown that the form of the hazard function, the proportion of cancer cases, the complexity of the model, and the sample size can all influence the accuracy of parameter estimation in multistate models. Therefore, we assess the performance of the methods discussed in the “Overview of Compared Methods” section through simulation studies based on the above factors. We employed a 2 ×2× 2 × 2 factorial design by varying the assumed parametric model of the progression times (x,t) , the proportion of observed state 3 events, the number of covariates ( p ) in the model, and the sample size n . All analyses were performed in the statistical software R, version 4.1.2.^43^

### Data-Generating Process and Implementation

For i=1,2,…,n individuals, we generated 2 sets of data for the progression times $\left(x_{i},t_{i}\right)$ in Figure 1: one set assuming that $\left(x_{i},t_{i}\right)$ follows a Weibull distribution, and another set assuming that $\left(x_{i},t_{i}\right)$ follows an exponential distribution. We chose Weibull and exponential distributions for $\left(x_{i},t_{i}\right)$ to represent time-dependent (i.e., since state entry) and time-independent hazards of progression, respectively, for both state 1-to-2 and state 2-to-3. These choices correspond to semi-Markov and time-homogeneous Markov models, respectively. To achieve this, we considered the following AFT models^37^:

(1) $$\begin{array}{c} \log\left(x_{i}\right)=\beta_{x,0}+\beta_{x,1}z_{1,i}+\beta_{x,2}z_{2,i}+\cdots +\beta_{x,p}z_{p,i}+\sigma_{x}\epsilon_{i},\\ \log\left(t_{i}\right)=\beta_{t,0}+\beta_{t,1}z_{1,i}+\beta_{t,2}z_{2,i}+\cdots +\beta_{t,p}z_{p,i}+\sigma_{t}\xi_{i}, \end{array}$$

where $\beta_{0}$ and $\beta_{p}$ are the intercept and regression (of order p ) parameters, respectively. The $\left(\sigma_{x},\sigma_{t}\right)$ and $\left(\epsilon_{i},\xi_{i}\right)$ represent the scale parameters and error terms (random variables), respectively. More specifically, we simulated the 2 sets of data from the models

(2) $$\begin{array}{c} \log\left(x_{i}\right)=3+\beta_{x,1}z_{1,i}+\beta_{x,2}z_{2,i}+0.2\epsilon_{i}\\ \log\left(t_{i}\right)=1.2+\beta_{t,1}z_{1,i}+\beta_{t,2}z_{2,i}+0.3\xi_{i} \end{array}$$

(3) $$\begin{array}{c} \log\left(x_{i}\right)=3+\beta_{x,1}z_{1,i}+\beta_{x,2}z_{2,i}+\epsilon_{i}\\ \log\left(t_{i}\right)=1.2+\beta_{t,1}z_{1,i}+\beta_{t,2}z_{2,i}+\xi_{i} \end{array}$$

where the error terms ( $\epsilon_{i},\xi_{i}$ ) follow an extreme value distribution, so that $\left(x_{i},t_{i}\right)$ are Weibull in model (2) with increasing hazards (since $\sigma_{x}=0.2$ , $\sigma_{t}=0.3$ are both less than 1), and exponentially in model (3), since here $\sigma_{x}=\sigma_{t}=1$ . In both models, we set the regression parameters $\beta_{x}=\beta_{t}=0$ for p=0 and $\beta_{x}=\beta_{t}={\left(0.5,0.5\right)}^{⊤}$ for p=2 with covariates $z_{1,i}\sim N\left(0,1\right)$ and $z_{2,i}\sim$ Bernoulli(0.5), corresponding to a continuous and a binary covariate, respectively. We varied the sample size n={1,000,2,000} . Our arbitrary choice of intercepts (3 for $x_{i}$ and 1.2 for $t_{i}$ ) in both models resulted in the median of $x_{i}$ being greater than that of $t_{i}$ . Specifically, when p=0 , the median of $x_{i}$ was 18.7, compared with 3.0 for $t_{i}$ ; for p=2 , the medians were 23.1 for $x_{i}$ and 3.6 for $t_{i}$ . These reflect settings (see Chen et al.^12^), where individuals, on average, tend to remain longer in state 1 than in state 2.

We varied the proportions of individuals in the data by considering 2 “strengths” of right censoring: medium and strong. Medium censoring corresponds to approximately 45% of individuals in state 2 and 20% in state 3, while strong censoring corresponds to about 30% and 10%, respectively. To achieve these targets, we compared the simulated progression times $\left(x_{i},t_{i}\right)$ with the visit times $v_{i}$ and then tuned both the parameters of the assumed visit time distribution and the mean time to right censoring of $x_{i}$ accordingly. We generated 100 datasets for each simulation setting and fitted the 3-state model (Figure 1) using the 6 methods described in the “Overview of Compared Methods” section. Full details on the data-generating process and implementation, including the design of visit schedules, parameter tuning of the visit time distribution to achieve the target event proportions, convergence diagnostics, and the choice of priors for the Bayesian-based methods, are provided in Supplement 2. Finally, for methods that require model specification for $\left(x_{i},t_{i}\right)$ , we assumed both exponential and Weibull models for smms and BayesTSM and 2-phase models for msm-phase and cthmm.

### Evaluation Criteria

To evaluate and compare the performance of the 6 methods in our simulation study, we computed the marginal CIF. For each method and simulation setting, we estimated the 10th, 50th (median), and 90th percentiles of the marginal CIF. The 10th percentile of the CIF of the progression time x , for example, represents the probability $P\left(x\leq \tilde{x}\right)=0.1$ at a specified time point $\tilde{x}$ . We reported the following metrics^44^: relative error, root mean squared error (rMSE), and the 95% coverage rate (CR) across the 100 simulated datasets. The relative error is defined as $\left(\hat{\phi }-\phi \right)/\phi$ , and the rMSE is defined as $\sqrt{\frac{1}{100}\sum_{j=1}^{100}{\left({\hat{\phi }}_{j}-\phi \right)}^{2}}$ , where ϕ represents the true percentile CIF value and $\hat{\phi }$ denotes the model-based estimate of the percentile CIF. The 95% CR represents the proportion of the 95% confidence intervals (CIs) or credible intervals (Crls) that contain ϕ . We considered the marginal CIF as our parameter of interest for comparison, as it is applicable to all methods regardless of covariate inclusion in the model. In addition, the 10th, 50th, and 90th percentiles of the marginal CIF were chosen to represent the median and the tails of the CIF curve, respectively. Finally, we also compared the marginal cumulative hazard functions across methods.

### Simulation Results

**Figure 3:**
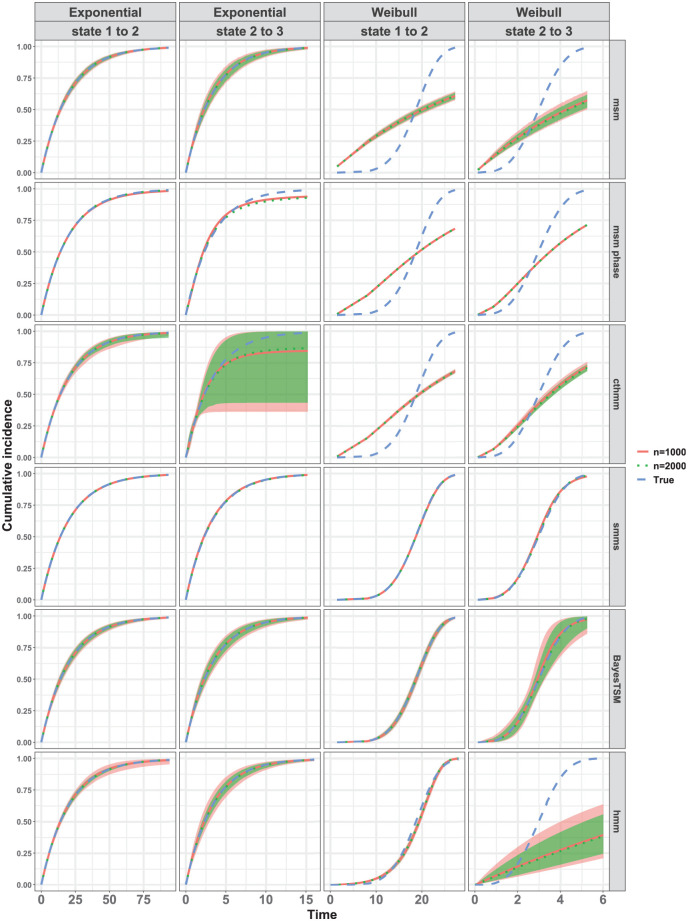
Marginal cumulative incidence functions (CIFs) compared for all 6 methods under strong censoring when p=0 for both exponential and Weibull models. The red and green lines represent the pointwise average of the 100 CIF estimates for samples sizes n=1,000 and n=2,000 , respectively; the blue line represents the true CIF. Healthy (state 1), cancer precursor (state 2), and cancer (state 3). The shaded regions indicate the 95% confidence or credible intervals, shown only for methods (msm, cthmm, BayesTSM, hmm) that provide functions to compute these intervals. For some methods, these shaded regions are very narrow and overlap almost entirely with the estimated or true CIFs (e.g., msm, state 1 to 2 under exponential model) and therefore may not be clearly distinguishable.

Figure 3 compares the true marginal CIF to the estimated marginal CIFs averaged pointwise across 100 datasets for n=1,000 and n=2,000 under the strong censoring setting with p=0 . All 6 methods were compared, assuming either a true exponential model or a true Weibull model for both state 1-to-2 and state 2-to-3 transitions. Results for other simulation conditions were similar (see Supplement 3). When examining the results under the true exponential model (Figure 3), the average CIFs for both n=1,000 and n=2,000 align well with the true CIF across all 6 methods, particularly for the state 1-to-2 transition. For the state 2-to-3 transition, results were similar. However, the estimated averaged CIFs from msm-phase and cthmm exhibited slight deviation from the true CIF, with the cthmm estimates showing notably wide confidence bounds. Under the Weibull model, only the estimated averaged CIFs from smms and BayesTSM appeared to align well with the true CIF for n=1,000 and n=2,000 in both transitions, as shown in Figure 3. The hmm implementation closely approximated the CIF for the state 1-to-2 transition but failed for the state 2-to-3 transition. Regarding msm, the results were as expected given that the method was specifically designed for Markov models, which assume an exponential distribution. For both transitions, the results from both cthmm and msm-phase indicate that, despite their semi-Markov assumptions, these methods failed to accurately approximate the true Weibull CIFs. This may be attributed to the specification of only a 2-phase model for the transient states in the 3-state model (Figure 1) and/or to the data-censoring structure considered in this article.

**Figure 4:**
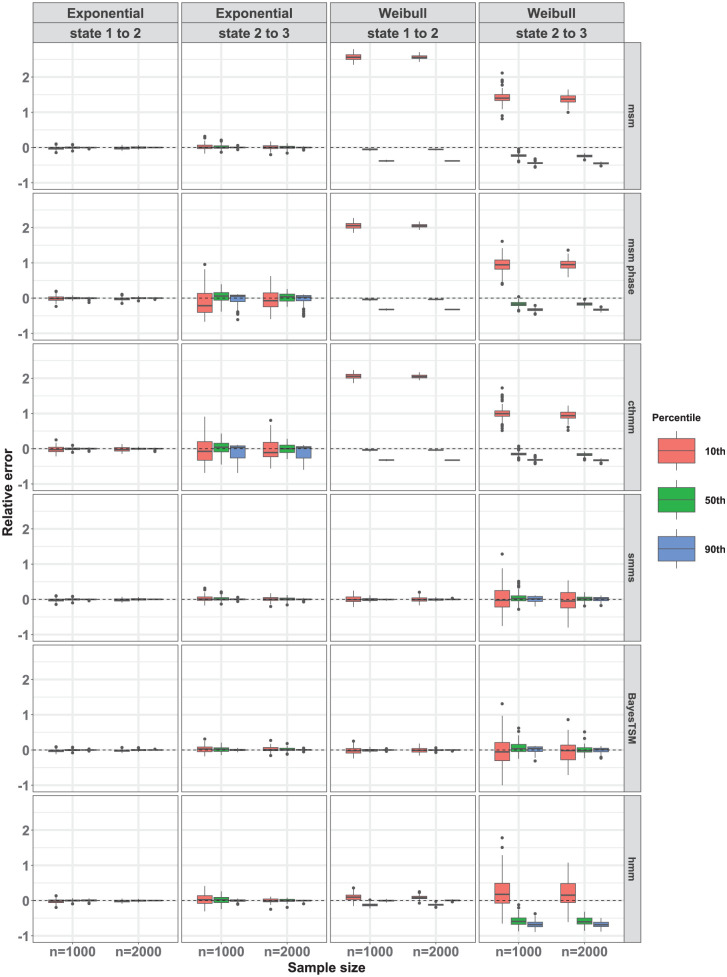
Relative error of the 10th, 50th, and 90th percentiles of the marginal cumulative incidence functions (CIFs). Compared for all 6 methods under strong censoring when p=0 for both exponential and Weibull models across 100 CIF estimates for samples sizes n=1,000 and n=2,000 . Healthy (state 1), cancer precursor (state 2), and cancer (state 3).

We further compared the performance of the methods by presenting box plots depicting the relative errors of the 10th, 50th (median), and 90th percentiles of the CIFs across 100 datasets in Figure 4. Results of other simulation conditions are reported in Supplement 3. We observe that under the true simulated exponential model for both transitions, all methods exhibited approximately unbiased estimates across all percentiles for both sample sizes n=1,000 and n=2,000 . However, estimates from the msm-phase and cthmm were stable for the state 1-to-2 transition but unstable for the state 2-to-3 transition, resulting in greater variability across the 100 estimates for this transition. This instability likely stems from weak parameter identifiability in the 2-phase model due to the sparsity of the data. Consistent with our previous observations under a true simulated Weibull model in Figure 3, only smms and BayesTSM exhibited nearly unbiased estimates across percentiles for both transitions and across sample sizes n . Furthermore, hmm exhibited nearly unbiased estimation across percentiles for the state 1-to-2 transition only. For cthmm, msm, and msm-phase, unbiased estimates were observed for the 50th percentile (i.e., median) of the CIFs for the state 1-to-2 transition but not for the 10th and 90th percentiles. Conversely, for the state 2-to-3 transition, these 4 methods (cthmm, hmm, msm, msm-phase) exhibited a substantial bias across all percentiles (Figure 4). The bias observed in the results of msm, for example, illustrates the bias that occurs when a semi-Markov process (e.g., Weibull) is modeled as a time-homogeneous Markov process, for which msm was designed. This contrasts with smms, cthmm, msm-phase, and BayesTSM, all of which assume semi-Markov models. In all cases of unbiased estimation, variance moderately decreased with sample size, as seen by the narrower interquartile ranges when n=2,000 .

Results showing the overall accuracy, measured by rMSE estimates, and the 95% CR estimates are reported in Supplement 3. For example, under the current simulation setting (i.e., p=0 and strong censoring), the rMSE estimates for both the state 1-to-2 and state 2-to-3 transitions were consistently low and similar across most methods under the exponential model, although estimates from msm-phase and cthmm were higher for the state 2-to-3 transition. Under the Weibull model, smms and BayesTSM yielded comparable estimates and achieved the lowest rMSEs for both the state 1-to-2 and state 2-to-3 transitions. Among the methods (msm, cthmm, BayesTSM, and hmm) that provide functions to compute 95% CIs or CRs, the resulting estimates showed good coverage overall, except for cthmm. For msm, BayesTSM, and hmm, most of the empirical CRs fell within the acceptable nominal range of 91% to 99%, as recommended by Burton et al.^45^ Finally, the results comparing the cumulative hazard functions (see Supplement 4) are consistent with those based on the CIFs.

In general, across all simulations, estimates of the relative error and the rMSE were lower under the medium censoring setting (see Supplement 3) compared with the strong censoring setting, particularly for the transition from state 2 to 3. This difference can be attributed to the lower proportion of individuals in state 3 (approximately 10%) compared with those under the medium censoring simulation setting (approximately 20%).

## Application to CRC Progression

In this section, we demonstrate the application of the selected methods outlined in the “Methods” section in modeling the progression to CRC using a surveillance dataset. The original dataset consists of n=836 individuals who underwent surveillance colonoscopy due to a family history of CRC in The Netherlands (see Supplement 5 for details). Only individuals who underwent at least 2 complete colonoscopies with no prior diagnosis of CRC or with no CRC at baseline colonoscopy were included. After applying these exclusion criteria, a total of 734 individuals remained in the dataset. Each colonoscopy provided information on the date and yield. Thus, during each surveillance visit, individuals were categorized into 1 of the 3 states: healthy, non-advanced adenoma (nAA; defined as 1–2 adenomas each <1 cm, without high-grade dysplasia or villous/tubulovillous histologic elements), and advanced neoplasia (AN; defined as either AA or CRC).

### Data Analysis

As an illustration, we fitted a model using only sex and birth year cohort as covariates. To include individuals who already have an adenoma at their first (i.e., baseline) colonoscopy in estimating the nAA-to-AN transition in a non-Markov model, it is necessary to account for the unknown time of entry into the nAA state. To address this, we adopted an approach similar to Kapetanakis et al.^46^ and Nevala et al.,^47^ using age as the time scale in our analysis, by assuming an age at which these individuals were considered healthy prior to their first colonoscopy, hereafter referred to as the “reference age” approach. Specifically, we assumed that all individuals were healthy at age 15 y motivated by the minimum age at first colonoscopy for individuals with an adenoma of any type in the data of 25 y. This assumption allows us to cover a broader time range over which progression hazards may be more likely to vary. All individuals were followed until they were either interval censored upon the detection of an event (nAA or AN) or right censored at their last surveillance date with no event detected. Similar to our simulation study, we estimated and compared the marginal CIFs (i.e., population-average risks) for each method. In addition, we computed HRs and the corresponding 95% Crls or CIs for covariates included in the model. Furthermore, we estimated the conditional CIFs for specific risk profiles based on the covariates included in the model (see Supplement 5). For each method, we used the same model specifications as those applied in our simulation study. Specifically, for the Bayesian methods, the specified priors (see Supplement 2 for details) serve primarily as regularization rather than encoding specific prior beliefs. These priors are intended to improve estimation stability in the presence of sparse data without biasing the parameter estimates, as demonstrated in Klausch et al.^14^ In principle, the methods are flexible with respect to the choice of priors and allow users to specify alternative priors. In addition, we checked for convergence of the estimates using the same approach as in our simulation study.

Finally, we assessed model fit for the state 1-to-2 transition by comparing the estimated CIFs from each method with the nonparametric maximum likelihood estimator (NPMLE)^48^ implemented in the R package survival.^49^

### Results

The characteristics of the 734 individuals included in the analysis at their first colonoscopy, categorized based on the findings at the time of censoring during surveillance, are presented in Supplement 5. These individuals were followed for a median of 6 y (interquartile range: 6–6 y) after the first colonoscopy. The mean age was 53 y. Among the n=734 individuals in the dataset, 447 (65%), 208 (28.3%), and 49 (6.7%) were classified as healthy (state 1), nAA (state 2), and AN (state 3), respectively, at the time of censoring. In addition, the median birth year was 1951 (interquartile range: 1947–1956). The dataset included 327 men (44.6%) and 407 women (55.4%).

**Figure 5:**
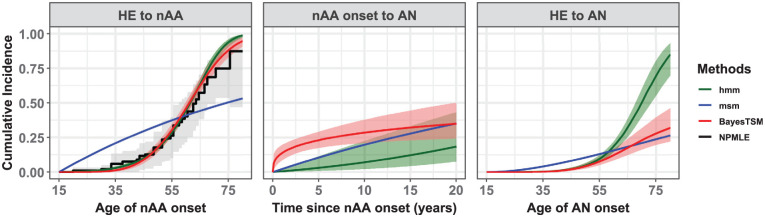
Estimated marginal cumulative incidence functions (CIFs) representing the population-average risks compared for msm, hmm, and BayesTSM based on a model with sex and birth year as variables. The third panel (i.e., HE to AN) is the sum of the transitions HE to nAA and nAA onset to AN. The CIF from the NPMLE was included solely to assess the model fit for the HE to nAA transition. Bootstrapped 95% confidence intervals of the NPMLE shown as gray-shaded region while the light-red shaded region corresponds to the 95% credible intervals from BayesTSM and the light-green shaded region to those from hmm. The estimates from smms, msm-phase, and cthmm were not included as they did not yield converged solutions.

The packages smms, msm-phase, and cthmm showed convergence problems during model estimation. Although various methods were attempted to resolve these issues (e.g., different starting values, stricter convergence limits), we could not obtain converged solutions. To the contrary, BayesTSM, hmm, and msm did yield converged solutions. Figure 5 compares the estimated marginal CIFs across converged methods. For the first transition, the estimates can be contrasted with the NPMLE. These estimates were similar between BayesTSM and hmm, consistent with the Weibull model results from our simulation study (Figure 3). The CIFs from both methods closely matched those from the NPMLE, with the 95% confidence bounds largely encompassing the estimates from both methods. This can be seen as a validation of model fit for BayesTSM and hmm. The narrower credible bounds seen in both methods compared with those from the NPMLE arise because they are based on parametric models that estimate fewer parameters than the NPMLE, resulting in lower variance. In contrast, the CIF estimates from msm differed significantly. For example, the nAA risk estimates at age 30 y were approximately 0 up to 3 decimal places for BayesTSM and hmm. However, for msm, the nAA risk was estimated at 16.2% (95% CI: 14.9%–17.8%). In addition, the estimated scale parameter $\sigma_{x}$ of the AFT model in BayesTSM was found to be 0.21 (95% Crl: 0.18–0.24), suggesting that the hazard of developing nAA from HE increases with time.

The estimated risks of developing AN after nAA onset varied across methods (middle panel, Figure 5). For example, the 5-y risk estimates were 23.1% (95% Crl: 17.4%–29.4%), 2.6% (95% Crl: 0.7%–10.7%), and 10.9% (95% CI: 8.1%–14.5%) for BayesTSM, hmm, and msm, respectively. The scale parameter $\sigma_{t}$ of the AFT model estimated using BayesTSM was 2.84 (95% Crl: 1.57–5.52), suggesting that the hazard of developing AN after nAA onset decreases with time. In addition, for the sum of the 2 transitions (i.e., HE to AN, third panel in Figure 5), the risk estimates for msm, hmm, and BayesTSM also showed notable differences, although the estimates from hmm and BayesTSM were similar up to about age 55 y.

Results showing the effect of sex and birth year on the hazards of developing nAA from HE and AN after nAA onset are given in Table 2. All 3 methods estimated higher transition rates from HE to nAA for males than females, with HRs of 1.18 (95% CI: 0.95–1.47) for msm, 1.16 (95% Crl: 0.93–1.45) for hmm, and 1.17 (95% CrI: 0.94–1.46); however, all intervals included 1, indicating no significant sex effect. In contrast, birth year showed a significant association with the hazard of developing nAA in both hmm (HR of 1.07, 95% Crl: 1.05–1.10) and BayesTSM (HR of 1.07, 95% CrI: 1.04–1.09), suggesting that individuals born more recently have a higher hazard of transitioning from HE to nAA. However, this was not the case for msm (HR of 0.99, 95% CI: 0.98–1.01). For the nAA-to-AN transition, males again had higher estimated hazards across all methods, with HRs of 1.25 (95% CI: 0.73–2.12) for msm, 1.38 (95% Crl: 0.81–2.37) for hmm, and 1.21 (95% Crl: 0.81–1.91) for BayesTSM; however, none of these were statistically significant. Birth year showed a significant effect for msm (HR of 1.05, 95% CI: 1.01–1.08) and hmm (HR of 1.07, 95% Crl: 1.02–1.12) but not for BayesTSM (HR of 1.01, 95% Crl: 0.96–1.04). Finally, given that BayesTSM outperformed both msm and hmm in simulations, we consider its results presented in this section to be the most reliable. Moreover, the 95% Crls for the Weibull AFT scale parameters ( $\sigma_{x},\sigma_{t}$ ) of both transitions from BayesTSM did not include 1, suggesting that a non-Markov model such as BayesTSM provided a better fit for both transitions.

**Table 2:** Effect of Sex and Birth Year on the Hazard of Developing nAA from HE and AN since nAA Onset

Transition	Method	Covariate	Estimate	HR	95% Cl
HE → nAA	msm	Sex: male (ref. female)	0.17	1.18	(0.95, 1.47)
		Birth year	−0.01	0.99	(0.98, 1.01)
	hmm	Sex: male (ref. female)	0.15	1.16	(0.93, 1.45)
		Birth year	0.07	1.07	(1.05, 1.10)
	BayesTSM	Sex: male (ref. female)	0.16	1.17	(0.94, 1.46)
		Birth year	0.07	1.07	(1.04, 1.09)
nAA → AN	msm	Sex: male (ref. female)	0.22	1.25	(0.73, 2.12)
		Birth year	0.05	1.05	(1.01, 1.08)
	hmm	Sex: male (ref. female)	0.32	1.38	(0.81, 2.37)
		Birth year	0.07	1.07	(1.02, 1.12)
	BayesTSM	Sex: male (ref. female)	0.19	1.21	(0.81, 1.91)
		Birth year	0.01	1.01	(0.96, 1.04)

HE, healthy; nAA: nonadvanced adenoma; AN: advanced neoplasia; Estimate: estimated regression coefficients; HR: hazard ratio; 95% Cl: 95% confidence or credible interval.

## Discussion

We presented 6 methods and their corresponding R software packages (msm, msm-phase, cthmm, smms, BayesTSM, and hmm) for fitting multistate models on the basis of cancer screening data where observations are censored after an intervention. The intervention refers to the removal of cancer precursors (referred to as a state 2 event), so that transition to cancer (referred to as a state 3 event) is prevented in these individuals.

In our simulation studies with time-independent hazards for both transitions (from state 1-to-2 and from state 2-to-3), all 6 methods performed well overall. The ability of the methods to correctly approximate the true risks, particularly for the unobserved state 2-to-3 transition, can be attributed to the memoryless property of exponential models, where the transition to state 3 is independent of the time spent in state 2. We also explored settings in which progression hazards are dependent on time since entry into a given state, as we did in the application section (cf. Figure 5). The 2-phase models assumed in msm-phase and cthmm showed poor performance, despite their semi-Markov assumptions. Increasing the number of phases might improve model fit, but it could lead to parameter identifiability issues due to the unobserved state 2 to 3 transition, as we encountered with cthmm. These issues were also noted by the developers of these methods.^18,30^ Despite their poor performance in the current study, these methods have been applied to screening and surveillance data with different censoring structures.^30,41,50,51^ We also aimed to illustrate the potential bias arising from model misspecification in progression risk estimation, which is crucial for optimizing cancer screening and surveillance frequency.^15,16,35^ Results based on msm demonstrated this by showing a substantial bias in risk estimates when the true hazards were assumed to be dependent on time since state entry. This bias was anticipated because msm, a widely used R software package for fitting multistate models to screening data (see Wei et al.,^52^ Kunst et al.,^53^ Meireles et al.,^54^ Moreira et al.,^55^ Sutradhar et al.,^56^ Sutradhar and Barbera,^57^ Taghipour et al.,^58^ Jia et al.^59^) assumes time-homogeneous Markov processes. This issue was also investigated in simulation studies by Aastveit et al.^29^ and Cheung et al.,^19^ both of whom observed bias in their analyses when a semi-Markov process was modeled using a Markov model. Despite this potential bias, previous studies have relied on Markov models for cancer risk estimation (see Yen et al.,^11^ Chen et al.,^12^ Cafferty et al.,^13^ Shen et al.,^26^ Nevala et al.,^47^ Wei et al.,^52^ Kunst et al.,^53^ Sutradhar et al.,^56^ Sutradhar and Barbera,^57^ Taghipour et al.,^58^ Jia et al.,^59^ Tian et al.^60^). In contrast, both *smms* and *BayesTSM* performed similarly well in approximating the true risk for both transitions in the simulations. The ability of these methods to correctly approximate the true risks that are dependent on time since state entry highlights their appropriateness for fitting multistate models to cancer screening data with censoring after intervention. However, when analyzing the real-world CRC surveillance data (“Application to Colorectal Cancer Progression” section), smms, msm-phase, and cthmm suffered from numerical optimization and convergence problems. Hence, despite smms performing well in simulations, its applicability to cancer screening data with censoring after intervention, as considered in this article, may be limited. These problems likely stem from a weakly identifiable likelihood, which is attributed to the sample size (i.e., n=734 ) and a low proportion (7%) of AN cases in the data, compared with the higher proportions (10% and 20%) used in the simulation study. Additional applications and evaluations would be beneficial to better understand its performance in this specific type of cancer screening data with censoring after intervention. Interestingly, when the sample size was doubled (i.e., n=1,468 ), these issues were resolved for the smms, msm-phase, and cthmm packages. This was also demonstrated in an additional simulation (see Supplement 4), in which the convergence rate for smms improved when the sample size was doubled from 734 to 1,468 . These results suggest that the size of the original dataset (i.e., n=734 ) may have contributed to these problems. In contrast, BayesTSM overcomes these problems by applying regularized parameter estimation using weakly informative priors.

We note that the use of semi-Markov models and hence the BayesTSM model, for modeling cancer screening or surveillance data, is particularly relevant when using the reference age approach, as we demonstrated in the application section. Age is a well-known variable associated with cancer risk, and the use of the reference age approach, in many situations (e.g. Kapetanakis et al.^46^), leads to the hazard rate varying with time since state entry. Similarly, in our case study, the nAA risk estimates were noticeably different for *msm* compared with *hmm* and *BayesTSM* , with the differences emerging most likely due to the time-homogeneity assumption by *msm* . A similar difference was observed by Nevala et al.^47^ in their CRC modeling study, in which estimates of the state occupancy probabilities obtained from a hidden Markov model using the reference age approach differed significantly from those produced by a Kaplan–Meier model. The similarity in the nAA risk estimates among NPMLE, *hmm* , and *BayesTSM* , in contrast to the estimates from *msm* , suggests that *msm* had the worst fit for the HE-to-nAA transition. In addition, the estimate of the AFT Weibull scale parameter $\sigma_{t}=$ 2.84 (95% CrI: 1.57–5.52) from BayesTSM strongly suggests that a nonexponential distribution provided a better fit for the transition nAA onset to AN.

Several directions for future research can be pursued. First, the current study evaluates the performance of selected methods under the assumption of no observation errors during surveillance. Second, we selected methods only applicable to a 3-state cancer model with interval-censored data and did not include death as a health state. Future studies should consider extending this work to include methods that account for observation errors during follow-up and incorporate more disease states into the model, such as death as a competing risk (see, e.g., Lange and Minin,^18^ Nevala et al.,^47^ Kang and Lagakos,^61^ Tan et al.,^62^ Brenner et al.,^63^ Bhattacharjee et al.^64^)

In conclusion, we demonstrated that the assumption regarding the time dependency of progression hazards between health states strongly affects the performance of existing methods for multistate cancer models using cancer screening data with censoring after intervention. Therefore, careful consideration is crucial when selecting a method, as an inappropriate choice can lead to biased parameter estimates and potentially misleading cancer screening and surveillance recommendations.

## Supplemental Material

sj-pdf-1-mdm-10.1177_0272989X261422681 – Supplemental material for A Comparison of Methods for Modeling Multistate Cancer Progression Using Screening Data with Censoring after Intervention Supplemental material, sj-pdf-1-mdm-10.1177_0272989X261422681 for A Comparison of Methods for Modeling Multistate Cancer Progression Using Screening Data with Censoring after Intervention by Eddymurphy U. Akwiwu, Veerle M. H. Coupé, Johannes Berkhof and Thomas Klausch in Medical Decision Making
