# Supplementary material for: A Comparison of Methods for Modeling Multistate Cancer Progression Using Screening Data with Censoring after Intervention
Source: Med Decis Making. 2026 Mar 13;46(5):575–90. doi: 10.1177/0272989X261422681 (PMC13242538; doi:10.1177/0272989X261422681)
Supplement: sj-pdf-1-mdm-10.1177_0272989X261422681 – Supplemental material for A Comparison of Methods for Modeling Multistate Cancer Progression Using Screening Data with Censoring after Intervention [file sj-pdf-1-mdm-10.1177_0272989X261422681.pdf]

Supplement to: A comparison of methods for modelling multi-state cancer progression using screening data with censoring after intervention

Contents

1 An example of cancer screening data with censoring after intervention **3**

2 Full details on the data generating process and implementation **5**

3 Additional results from the simulation study **8**

3.1 Marginal cumulative incidence functions plots under strong censoring when $p = 2$ . . 8

3.2 Marginal cumulative incidence functions plots under medium censoring when $p = 0$ 9

3.3 Marginal cumulative incidence functions plots under medium censoring when $p = 2$ 10

3.4 Box plots of relative errors under strong censoring when $p = 2$ 11

3.5 Box plots of relative errors under medium censoring when $p = 0$ 12

3.6 Box plots of relative errors under medium censoring when $p = 2$ 13

3.7 Table showing root mean squared errors of exponential and Weibull models under strong censoring when $p = 0$ and $p = 2$ 14

3.8 Table showing root mean squared errors of exponential and Weibull models under medium censoring when $p = 0$ and $p = 2$ 15

3.9 Table showing the 95% coverage rates for the marginal percentiles of the cumulative incidence function for exponential and Weibull models under strong censoring when $p = 0$ and $p = 2$ 16

3.10 Table showing the 95% coverage rates for the marginal percentiles of the cumulative incidence function for exponential and Weibull models under medium censoring when $p = 0$ and $p = 2$ 17

4 Additional simulation study **18**

4.1 `smms` vs. `BayesTSM` 18

4.2 Cumulative hazard functions 18

5 An application to colorectal cancer progression **23**

5.1 Characteristics of the 734 individuals included in the analysis 23

5.2 Results of the conditional cumulative incidence function using BayesTSM package 23

1 An example of cancer screening data with censoring after intervention

Table S1 presents an example of cancer screening data with censoring after intervention from a hypothetical screening or surveillance program. This example includes five visit times (e.g., in years) that follow the observation process depicted in Figure 2 of the main manuscript, the resulting censored health state δ (i.e., event type), and individual characteristics such as sex and age. For individuals 1, 4, 6, and 7, no cancer precursor or cancer was detected during their respective visit times; therefore, they were classified as $\delta = 1$ and right-censored at their respective last visits. Thus, their respective right-censored data are $(6, \infty)$, $(11, \infty)$, $(5, \infty)$, and $(7.5, \infty)$. Additionally, individual 2 had no event (i.e., a cancer precursor or cancer) detected during the first visit at $v_1 = 2.5$ years but was found to have a cancer precursor at $v_2 = 8$ years. Consequently, this individual was classified as $\delta = 2$ and was considered interval-censored at $(2.5, 8]$, with subsequent visits disregarded. Similarly, individuals 3 and n, also classified as $\delta = 2$, were interval-censored at $(8.5, 10.5]$ and $(13, 16.5]$, respectively. Finally, for individual 5, no event was detected during the first four visits, but cancer was identified at $v_5 = 13.5$ years. Consequently, this individual was classified as $\delta = 3$ and was considered interval-censored at $(10, 13.5]$. Similarly, individual 8, also classified as $\delta = 3$, was interval-censored data at $(0, 6]$. Furthermore, this individual is said to be left-censored (a special case of interval-censored data) since an event (here, cancer) was detected at the first visit after baseline.

In general, most individuals in the example dataset were classified as $\delta = 1$, followed by those classified as $\delta = 2$, with the fewest individuals classified as $\delta = 3$. This distribution of events at the time of censoring, as well as the randomly distributed visit times, is typical for cancer screening data with censoring after intervention (see, e.g., (1–4)).

Table S1. A hypothetical example of a cancer screening data with censoring after intervention, illustrating the three possible event types (δ) after baseline ($v_0 = 0$) across five screening or surveillance visit times (in years) for $i = 1, 2, \cdots, n$ individuals.

	Visit times							
Individual i	v_1	v_2	v_3	v_4	v_5	δ	Sex	Age
1	2	4.5	6	∞	-	1	Male	51
2	2.5	8	-	-	-	2	Male	50
3	4	6	8.5	10.5	-	2	Female	59
4	3.5	6.5	8	11	∞	1	Male	67
5	2	5.5	7	10	13.5	3	Female	62
6	5	∞	-	-	-	1	Male	70
7	3	7.5	∞	-	-	1	Female	52
8	6	-	-	-	-	3	Male	55
.	.	.	.	.	.	.	.	.
.	.	.	.	.	.	.	.	.
.	.	.	.	.	.	.	.	.
n	4	5.5	8	13	16.5	2	Male	60

$\delta \in \{1, 2, 3\}$ correspond to healthy, cancer precursor and cancer states, respectively.

– = no observation made.

2 Full details on the data generating process and implementation

For $i = 1, 2, \ldots, n$ individuals, we generated two sets of data for the progression times (x_i, t_i) in Figure 1 of the main manuscript: one set assuming that (x_i, t_i) follows a Weibull distribution, and another set assuming that (x_i, t_i) follows an exponential distribution. We chose Weibull and exponential distributions for (x_i, t_i) to represent time-dependent (i.e., since state entry) and time-independent hazards of progression, respectively, for both state 1 to 2 and state 2 to 3. These choices correspond to semi-Markov and Markov models, respectively. To achieve this, we considered the following AFT models (5):

$$\begin{aligned} \log(x_i) &= \beta_{x,0} + \beta_{x,1} z_{1,i} + \beta_{x,2} z_{2,i} + \cdots + \beta_{x,p} z_{p,i} + \sigma_x \epsilon_i, \\ \log(t_i) &= \beta_{t,0} + \beta_{t,1} z_{1,i} + \beta_{t,2} z_{2,i} + \cdots + \beta_{t,p} z_{p,i} + \sigma_t \xi_i, \end{aligned} \tag{1}$$

where β_0 and β_p are the intercept and regression (of order p) parameters, respectively. The (σ_x, σ_t) and (ϵ_i, ξ_i) represent the scale parameters and error terms (random variables), respectively. More specifically, we simulated the two sets of data from the models

$$\begin{aligned} \log(x_i) &= 3 + \beta_{x,1} z_{1,i} + \beta_{x,2} z_{2,i} + 0.2\epsilon_i \\ \log(t_i) &= 1.2 + \beta_{t,1} z_{1,i} + \beta_{t,2} z_{2,i} + 0.3\xi_i \end{aligned} \tag{2}$$

$$\begin{aligned} \log(x_i) &= 3 + \beta_{x,1} z_{1,i} + \beta_{x,2} z_{2,i} + \epsilon_i \\ \log(t_i) &= 1.2 + \beta_{t,1} z_{1,i} + \beta_{t,2} z_{2,i} + \xi_i \end{aligned} \tag{3}$$

where the error terms (ϵ_i, ξ_i) follow an extreme value distribution, so that (x_i, t_i) are Weibull in model (2) with increasing hazards (since $\sigma_x = 0.2$, $\sigma_t = 0.3$ are both less than 1), and exponentially in model (3), since here $\sigma_x = \sigma_t = 1$. In both models, we set the regression parameters $\beta_x = \beta_t = 0$ for $p = 0$ and $\beta_x = \beta_t = (0.5, 0.5)^\top$ for $p = 2$ with covariates $z_{1,i} \sim N(0, 1)$ and $z_{2,i} \sim \text{Bernoulli}(0.5)$, corresponding to a continuous and a binary covariate, respectively. We varied the sample size $n = \{1000, 2000\}$. Our arbitrary choice of intercepts (3 for x_i and 1.2 for t_i) in both models resulted in the median of x_i being greater than that of t_i. Specifically, when $p = 0$, the median of x_i was 18.7, compared to 3.0 for t_i; for $p = 2$, the medians were 23.1 for x_i and 3.6 for t_i. These reflect settings (see, e.g., (3)) where individuals, on average, tend to remain longer in state 1 than in state 2.

We considered two 'strengths' of right-censoring (medium and strong) which influences the proportion of state 2 and 3 events in the data. The visit times $\boldsymbol{v}_i = (v_{1,i}, v_{2,i}, \cdots, v_{m-1,i}, v_{m,i})$ during screening or surveillance for each individual i follow a similar process as in Figure 2 of the main manuscript, where the number of visits m varies across individuals. The first visit time after baseline, $v_{1,i}$, was drawn from a Uniform distribution $U(c_{min}, c_{max})$. Subsequent visit times were generated as $v_{j+1,i} \sim \text{Uniform}(v_{j,i} + c_{min}, v_{j,i} + c_{max})$ for $j = 1, 2, \cdots, m$ until $v_{j+1,i} > v_{rc,i}$. Here, $v_{rc,i} \sim \exp(\theta^{-1})$ represents the time at which an individual is right-censored, either due to loss to follow-up or end of surveillance period. The parameter θ indicates the mean time to right-censoring of the first progression time x_i whereas (c_{min}, c_{max}) can be seen as the minimum and maximum time between two consecutive visits during surveillance. Thus, the parameters $(\theta, c_{min}, c_{max})$ determine the distribution of the observed health states in the data given the progression times (x_i, t_i).

Given the progression times (x_i, t_i) and the visit times $\boldsymbol{v}_i$, the right-censored data $(v_{m,i}, \infty)$ for $\delta_i = 1$ (state 1) and the interval-censored data $(v_{m-1,i}, v_{m,i}]$ for $\delta_i \in \{2, 3\}$ (states 2 and 3), as described in Section 2.1 of the main manuscript, were determined as follows

$$\delta_i = \begin{cases} 1 & \text{if } v_{m,i} < x_i < \infty \\ 2 & \text{if } v_{m-1,i} < x_i \leq v_{m,i} < x_i + t_i \\ 3 & \text{if } v_{m-1,i} < x_i < x_i + t_i \leq v_{m,i} < \infty. \end{cases} \quad (4)$$

As mentioned earlier, we considered two 'strengths' of right-censoring (medium and strong), such that the proportion of individuals in state 2 and 3 varied in the dataset. To achieve this, we manually tuned the parameters $(\theta, c_{min}, c_{max})$ in a large population setting of $n = 10^6$ individuals, regardless of the assumed probability distribution for (x_i, t_i) in the model. This ensured that the distribution of the event types $\delta_i \in \{1, 2, 3\}$ was approximately the same in both settings with $p = 0$ and $p = 2$ covariates. The chosen values of the parameters $(\theta, c_{min}, c_{max})$ and the resulting distribution of $\delta_i \in \{1, 2, 3\}$ are reported in Table S2. Strong right-censoring resulted in approximately 30% of the individuals in state 2 and 10% in state 3, whereas medium right-censoring resulted in approximately 45% of the individuals in state 2 and 20% in state 3.

Table S2. Parameter settings for the censoring parameters $(c_{min}, c_{max}, \theta)$ and the resulting distributions of δ_i under exponential and Weibull models for $p = 0$ and $p = 2$.

	Exponential model				Weibull model			
	Medium censoring		Strong censoring		Medium censoring		Strong censoring	
	$p = 0$	$p = 2$	$p = 0$	$p = 2$	$p = 0$	$p = 2$	$p = 0$	$p = 2$
c_{min}	1	1	1	1	1	1	1	1
c_{max}	3.8	4.0	3	3	6.7	7.3	6.0	5.5
θ	36.5	49	14	17.5	42	57.5	20	25.9
$\Pr(\delta_i = 1 \mid c_{min}, c_{max}, \theta)^\dagger$	0.355	0.354	0.589	0.590	0.352	0.355	0.594	0.591
$\Pr(\delta_i = 2 \mid c_{min}, c_{max}, \theta)^\dagger$	0.446	0.450	0.305	0.304	0.441	0.440	0.302	0.305
$\Pr(\delta_i = 3 \mid c_{min}, c_{max}, \theta)^\dagger$	0.199	0.196	0.106	0.107	0.206	0.205	0.104	0.103

$\delta_i \in \{1, 2, 3\}$ represent healthy (state 1), cancer precursor (state 2) and cancer (state 3).
Note: The parameters c_{min} and c_{max} represent the minimum and maximum time between two consecutive surveillance visits during surveillance, while θ denotes the mean time to right censoring of the first progression time x_i.
† Estimated in a large population setting of $n = 10^6$.

We generated 100 datasets for each simulation setting and fitted the three-state model (Figure 1 of the main manuscript) using the six methods described in Section 2.3 of the main manuscript. For the ML-based methods (`msm, msm-phase, cthmm, smms`), three different random starting values per dataset were initialized to ensure convergence to the global maximum and we evaluated the Hessian matrix to ensure that the estimated parameters are at a maximum. If non-convergence occurred for a run, we replaced the non-converged run with an additional run. In the case of the Bayesian-based methods (`hmm, BayesTSM`), we ran three randomly initialized chains of 10^5 iterations each. For `BayesTSM`, following (1), Student-t priors with 4 degrees of freedom were assigned to $(\beta_0, \beta_1, \beta_2)$, and half-normal $N^+(0, \sqrt{10})$ priors to the σ parameters. These weakly informative priors imply that, with 95% probability, the β parameters for a standardized covariate lie within $(-2.776, 2.776)$, or equivalently, the $\exp(\beta)$ lie between 0.062 and 16.12, allowing for large effects, while the σ parameters lie within $(0.099, 7.088)$. For `hmm`, normal priors $N(0, 20)$ were used, implying a 95% probability interval of approximately $(-39.20, 39.20)$ for the model parameters. We assessed for convergence according to suggestions by Gelman et al. (6) as follows. We ran the chains until the Gelman-Rubin convergence statistic $\hat{R}$ was below 1.1, and the effective sample size of posterior draws was at least 30 for each model parameter. For computational efficiency, the MCMC sampling was set to stop after a maximum of 2×10^6 iterations. If non-convergence

occurred afterwards, we replaced the chains by a new simulation run. We discarded half of the iterations as a burn-in period before calculating statistics. The same 100 datasets, generated based on a simulation setting (e.g., $n = 1000, p = 0$, strong censoring, Weibull models for (x_i, t_i)), were used for all six methods to ensure comparability. For methods that require model specification for (x_i, t_i), we assumed both exponential and Weibull models for `smms` and `BayesTSM`, and two-phase models for `msm-phase` and `cthmm`.

3 Additional results from the simulation study

3.1 Marginal cumulative incidence functions plots under strong censoring when $p = 2$

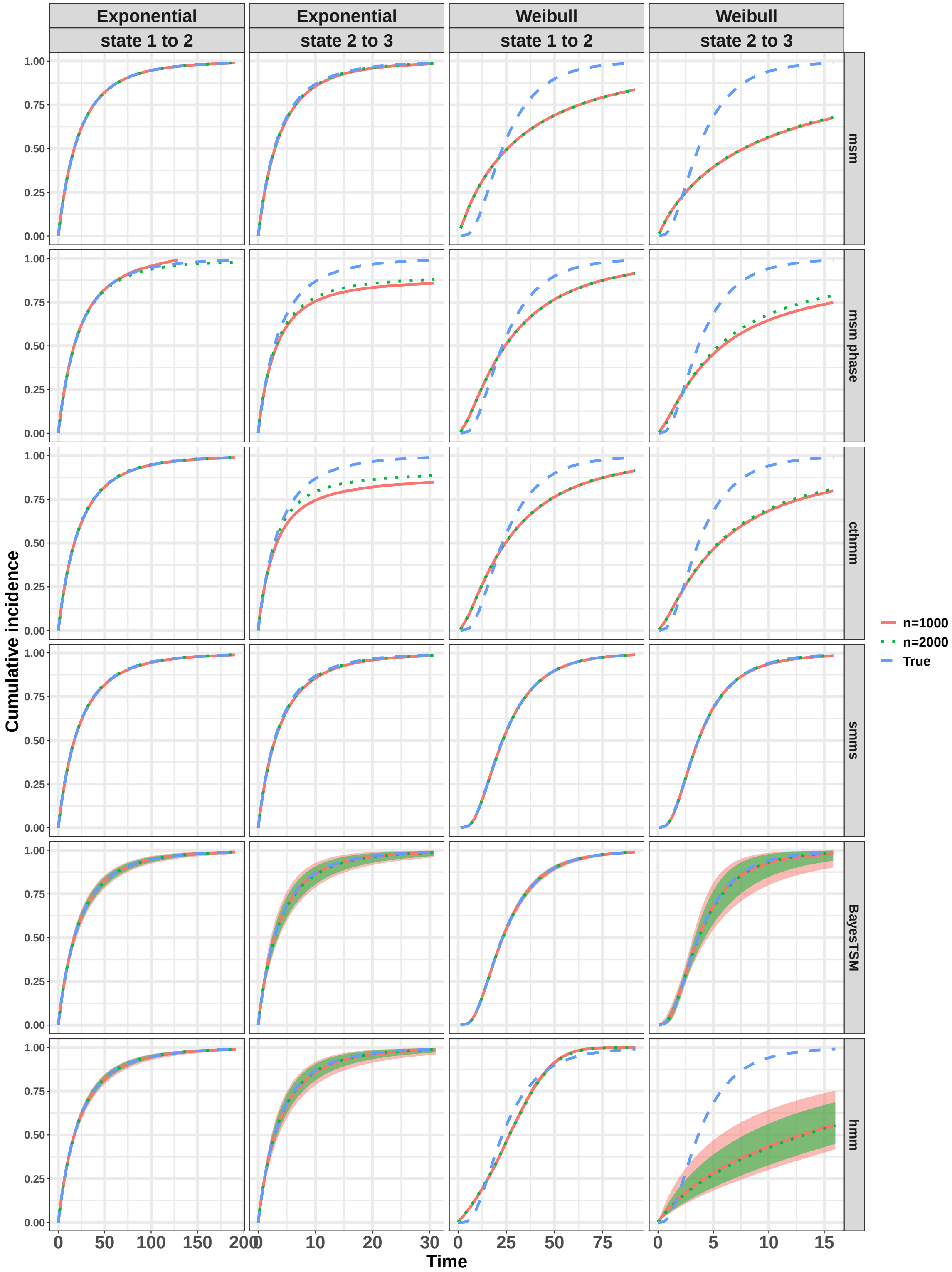

Figure S1. Marginal cumulative incidence functions (CIFs) compared for all six methods under strong censoring when $p = 2$.

3.2 Marginal cumulative incidence functions plots under medium censoring when $p = 0$

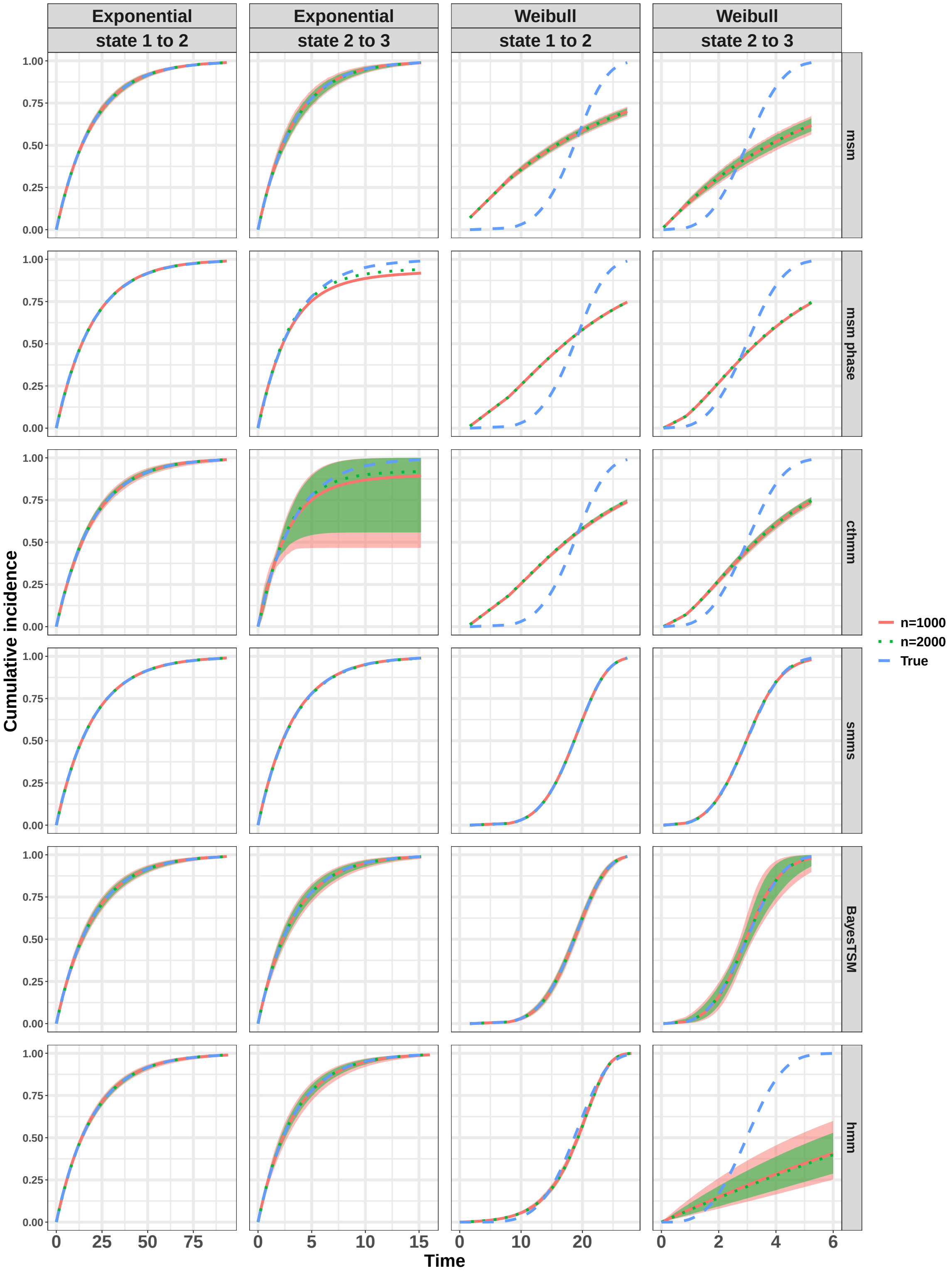

Figure S2. Marginal cumulative incidence functions (CIFs) compared for all six methods under medium censoring when $p = 0$.

3.3 Marginal cumulative incidence functions plots under medium censoring when $p = 2$

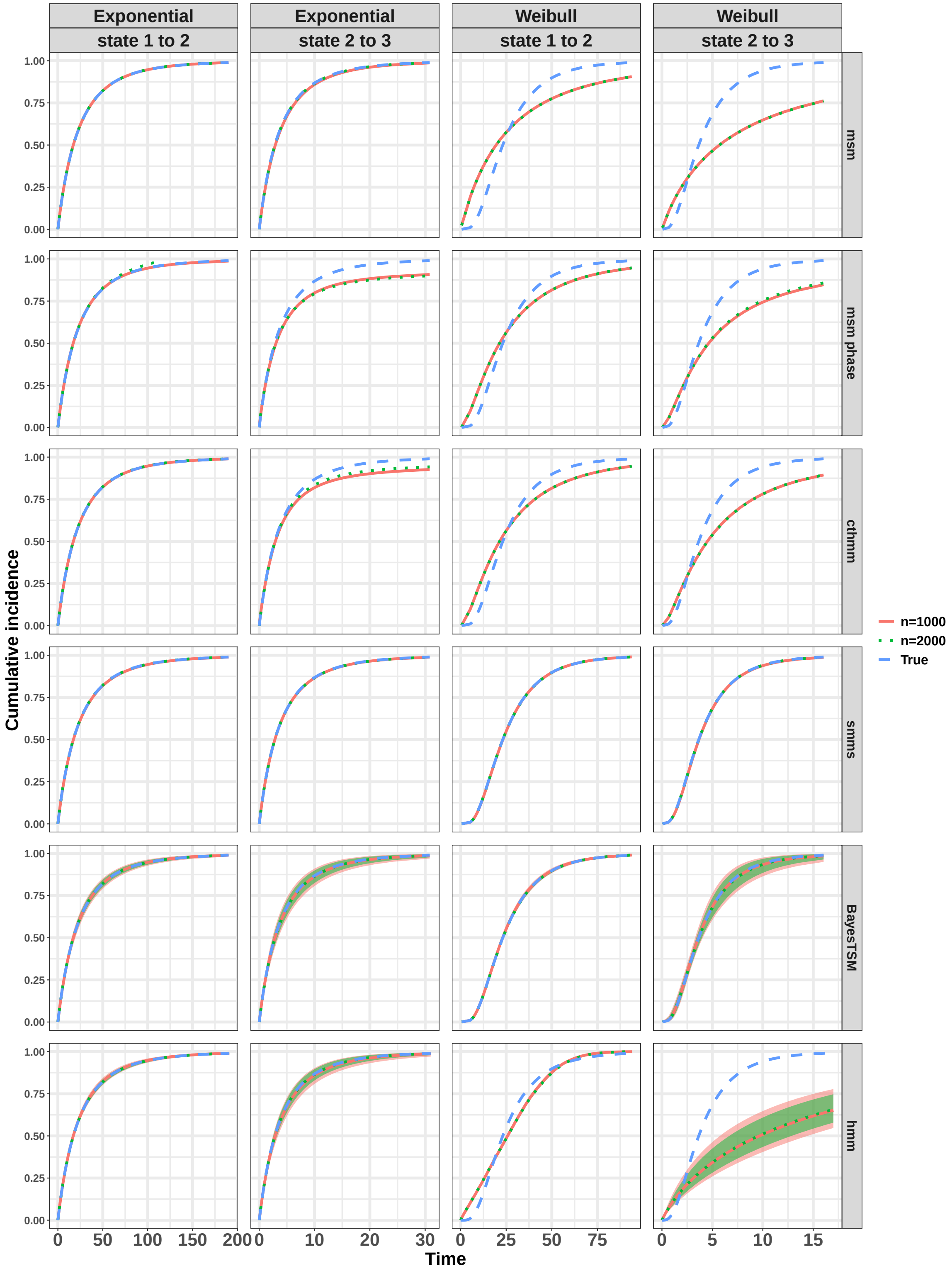

Figure S3. Marginal cumulative incidence functions (CIFs) compared for all six methods under medium censoring when $p = 2$.

3.4 Box plots of relative errors under strong censoring when $p = 2$

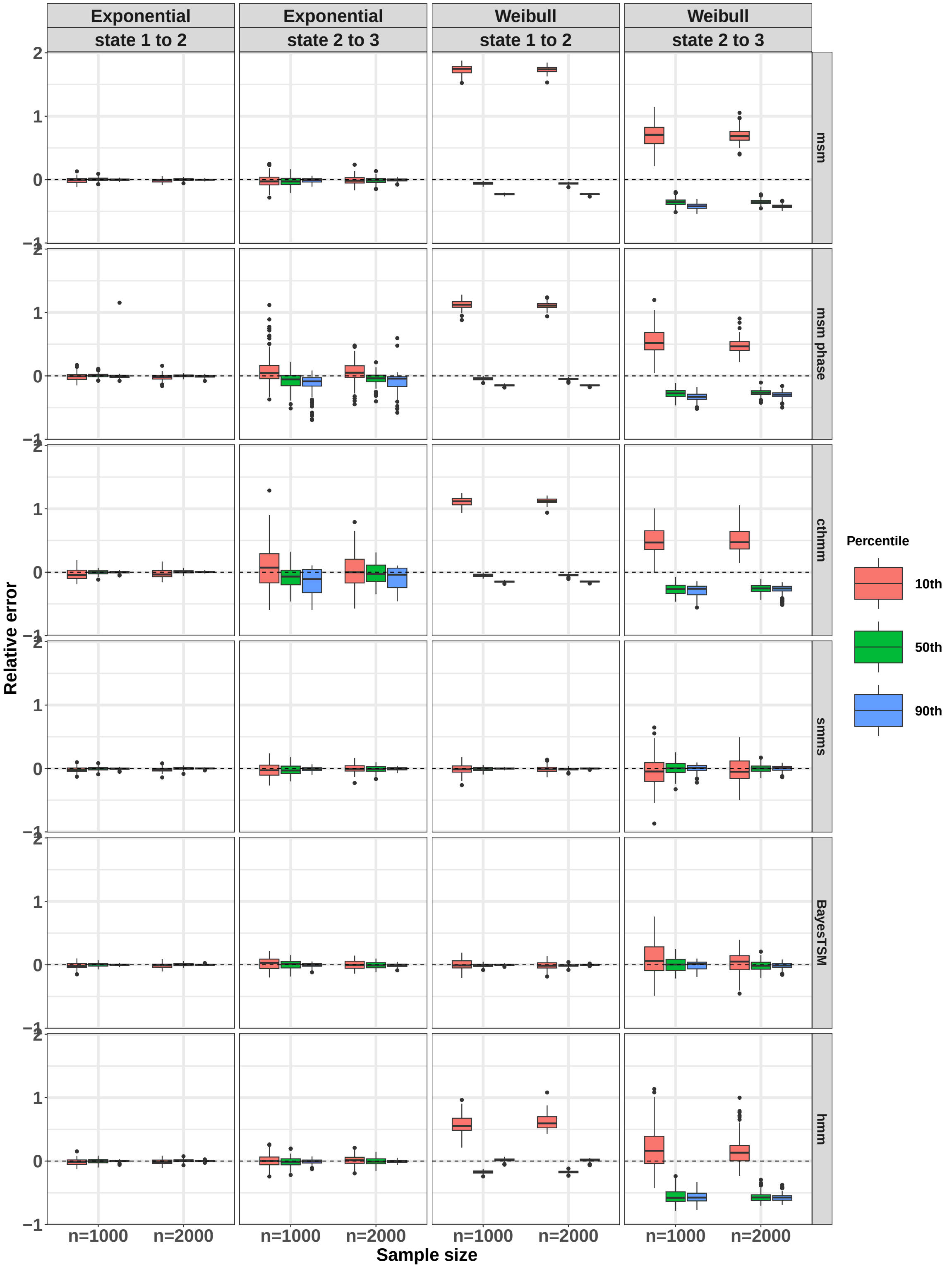

Figure S4. Relative error compared for all six methods under strong censoring when $p = 2$.

3.5 Box plots of relative errors under medium censoring when $p = 0$

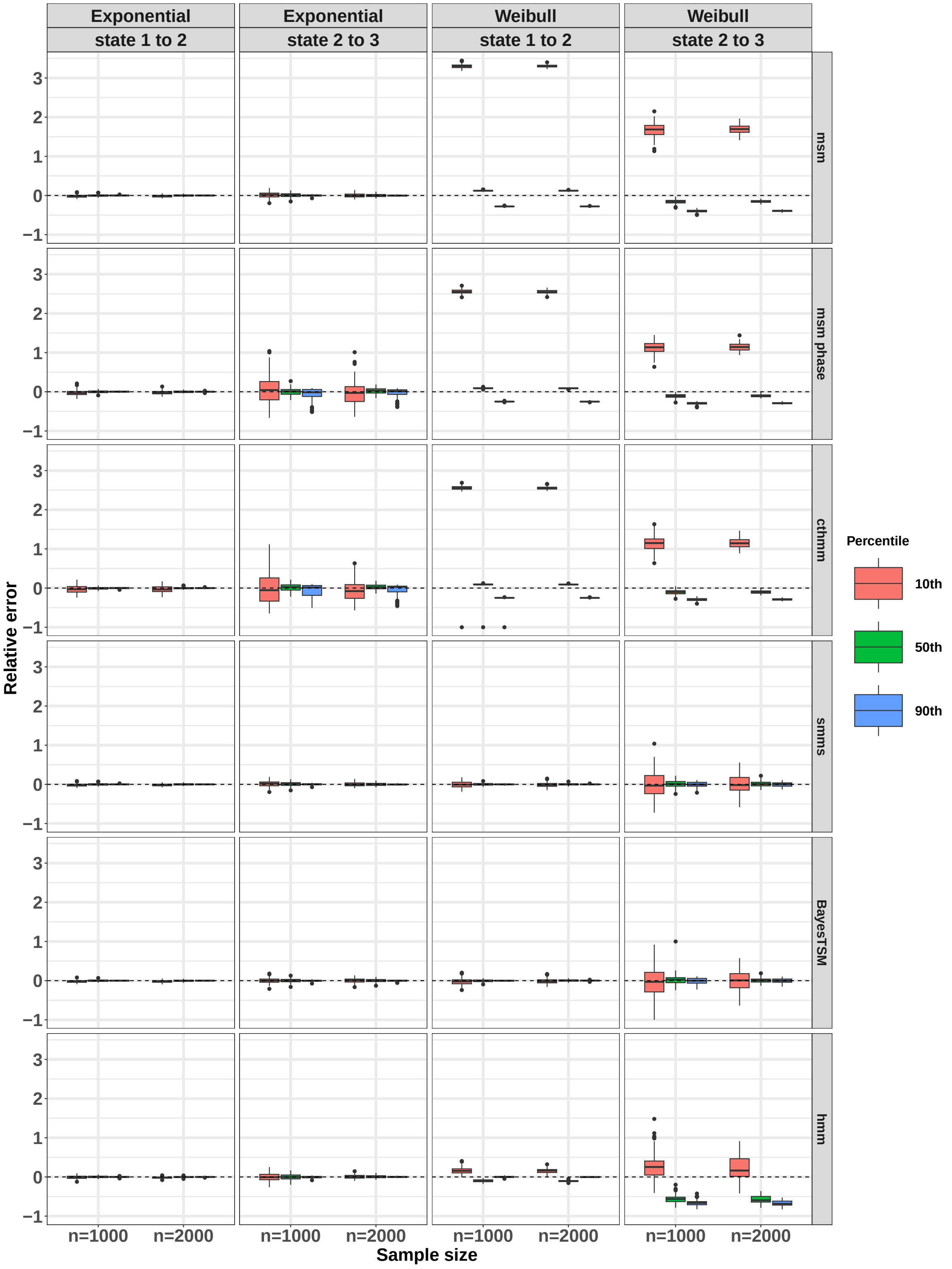

Figure S5. Relative error compared for all six methods under medium censoring when $p = 0$.

3.6 Box plots of relative errors under medium censoring when $p = 2$

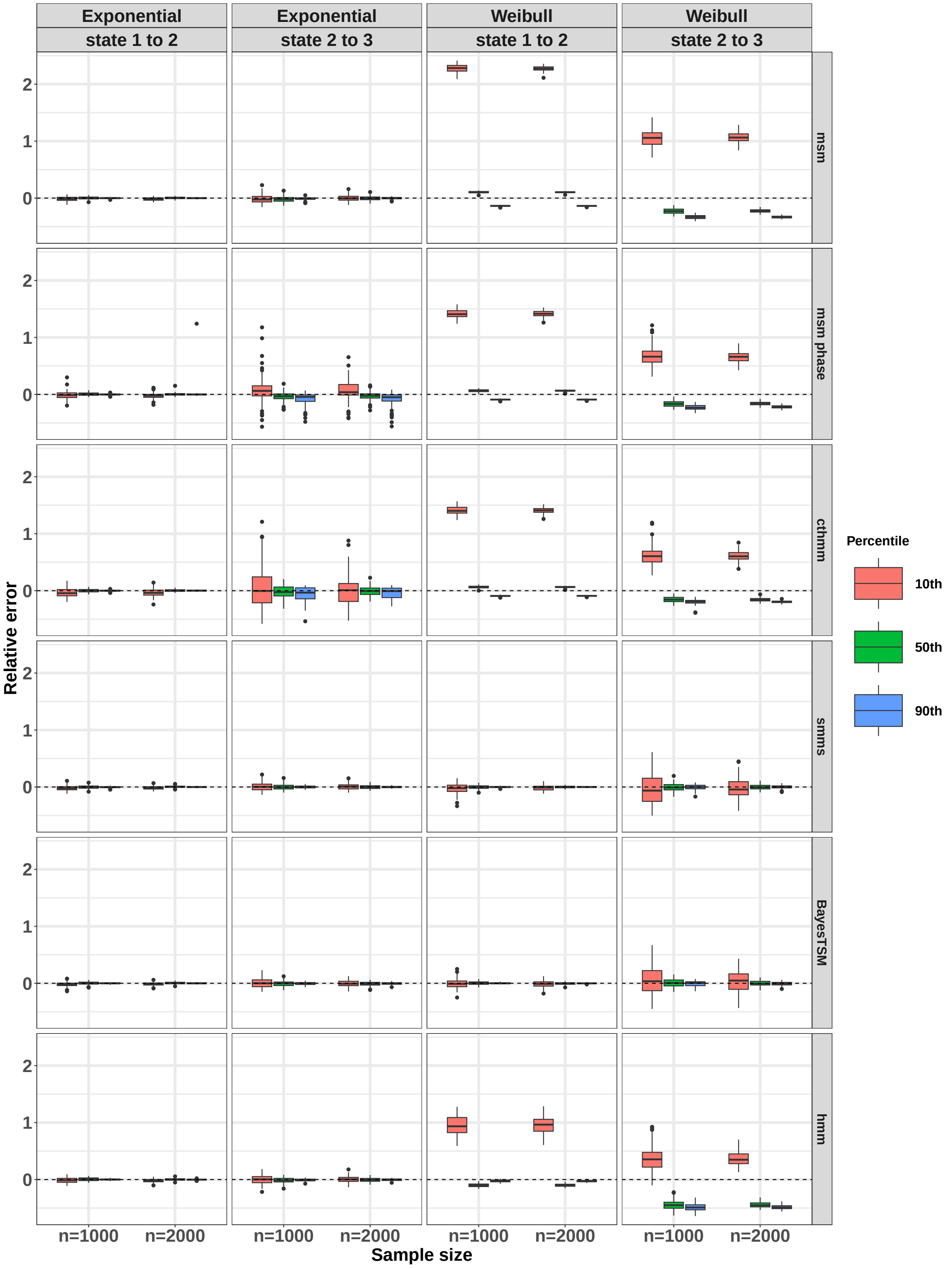

Figure S6. Relative error compared for all six methods under medium censoring when $p = 2$.

3.7 Table showing root mean squared errors of exponential and Weibull models under strong censoring when $p = 0$ and $p = 2$

Table S3. Comparing root mean squared errors of exponential and Weibull models under strong censoring when $p = 0$ and $p = 2$.

			$p = 0$				$p = 2$			
			state 1 → state 2		state 2 → state 3		state 1 → state 2		state 2 → state 3	
Method	Sample size	Percentile	Exponential	Weibull	Exponential	Weibull	Exponential	Weibull	Exponential	Weibull
msm	$n = 1000$	10th	0.005	0.256	0.009	0.143	0.005	0.174	0.011	0.072
		50th	0.017	0.029	0.032	0.120	0.015	0.032	0.042	0.179
		90th	0.011	0.342	0.020	0.401	0.012	0.209	0.036	0.383
	$n = 2000$	10th	0.004	0.256	0.007	0.137	0.003	0.173	0.007	0.071
		50th	0.013	0.027	0.025	0.124	0.010	0.032	0.027	0.177
		90th	0.009	0.343	0.017	0.407	0.009	0.209	0.023	0.379
msm-phase	$n = 1000$	10th	0.008	0.205	0.040	0.098	0.007	0.112	0.028	0.058
		50th	0.017	0.022	0.083	0.092	0.018	0.026	0.077	0.144
		90th	0.022	0.291	0.148	0.299	0.106	0.134	0.195	0.305
	$n = 2000$	10th	0.005	0.205	0.030	0.096	0.006	0.111	0.018	0.049
		50th	0.012	0.020	0.066	0.088	0.011	0.026	0.055	0.135
		90th	0.013	0.291	0.147	0.297	0.018	0.134	0.161	0.275
cthmm	$n = 1000$	10th	0.009	0.206	0.037	0.102	0.009	0.112	0.040	0.055
		50th	0.017	0.021	0.087	0.081	0.017	0.027	0.094	0.143
		90th	0.022	0.290	0.230	0.287	0.016	0.136	0.223	0.285
	$n = 2000$	10th	0.007	0.205	0.030	0.095	0.007	0.112	0.029	0.054
		50th	0.011	0.020	0.064	0.089	0.012	0.025	0.079	0.136
		90th	0.015	0.291	0.200	0.298	0.011	0.134	0.176	0.272
smms	$n = 1000$	10th	0.005	0.010	0.009	0.039	0.005	0.008	0.011	0.026
		50th	0.017	0.020	0.032	0.074	0.016	0.016	0.042	0.054
		90th	0.011	0.015	0.020	0.076	0.014	0.011	0.035	0.060
	$n = 2000$	10th	0.004	0.007	0.007	0.028	0.004	0.006	0.007	0.020
		50th	0.014	0.015	0.025	0.038	0.011	0.012	0.026	0.033
		90th	0.009	0.011	0.017	0.057	0.008	0.007	0.023	0.038
BayesTSM	$n = 1000$	10th	0.005	0.009	0.010	0.045	0.005	0.008	0.010	0.028
		50th	0.016	0.018	0.037	0.081	0.016	0.014	0.034	0.057
		90th	0.011	0.012	0.024	0.089	0.013	0.010	0.028	0.069
	$n = 2000$	10th	0.004	0.007	0.007	0.030	0.004	0.006	0.007	0.017
		50th	0.013	0.012	0.027	0.053	0.012	0.011	0.027	0.039
		90th	0.008	0.009	0.017	0.068	0.009	0.007	0.026	0.044
hmm	$n = 1000$	10th	0.006	0.013	0.014	0.054	0.005	0.057	0.009	0.038
		50th	0.017	0.066	0.050	0.296	0.017	0.089	0.040	0.289
		90th	0.027	0.020	0.028	0.609	0.013	0.028	0.035	0.516
	$n = 2000$	10th	0.003	0.011	0.005	0.038	0.003	0.061	0.007	0.025
		50th	0.012	0.062	0.022	0.300	0.012	0.088	0.030	0.293
		90th	0.008	0.014	0.016	0.615	0.009	0.024	0.025	0.520

Note: $p = 0$, no covariate; $p = 2$, two covariates.

3.8 Table showing root mean squared errors of exponential and Weibull models under medium censoring when $p = 0$ and $p = 2$

Table S4. Comparing root mean squared errors of exponential and Weibull models under medium censoring when $p = 0$ and $p = 2$.

			$p = 0$				$p = 2$			
			state 1 → state 2		state 2 → state 3		state 1 → state 2		state 2 → state 3	
Method	Sample size	Percentile	Exponential	Weibull	Exponential	Weibull	Exponential	Weibull	Exponential	Weibull
msm	$n = 1000$	10th	0.004	0.330	0.007	0.169	0.004	0.228	0.007	0.107
		50th	0.013	0.061	0.026	0.082	0.012	0.052	0.026	0.116
		90th	0.009	0.251	0.017	0.359	0.009	0.124	0.023	0.300
	$n = 2000$	10th	0.003	0.331	0.005	0.170	0.004	0.227	0.005	0.107
		50th	0.010	0.061	0.017	0.078	0.009	0.052	0.018	0.114
		90th	0.007	0.251	0.012	0.355	0.007	0.124	0.015	0.300
msm-phase	$n = 1000$	10th	0.007	0.256	0.038	0.113	0.007	0.142	0.025	0.071
		50th	0.015	0.046	0.049	0.061	0.014	0.034	0.043	0.087
		90th	0.012	0.225	0.143	0.267	0.011	0.083	0.124	0.210
	$n = 2000$	10th	0.005	0.256	0.029	0.115	0.005	0.141	0.020	0.066
		50th	0.010	0.046	0.036	0.054	0.010	0.033	0.038	0.082
		90th	0.009	0.225	0.105	0.262	0.008	0.082	0.137	0.198
cthmm	$n = 1000$	10th	0.010	0.255	0.039	0.115	0.009	0.142	0.038	0.063
		50th	0.051	0.068	0.048	0.060	0.015	0.034	0.052	0.082
		90th	0.013	0.241	0.163	0.265	0.012	0.083	0.129	0.179
	$n = 2000$	10th	0.008	0.256	0.027	0.116	0.008	0.141	0.027	0.061
		50th	0.011	0.046	0.036	0.054	0.010	0.032	0.041	0.080
		90th	0.008	0.225	0.128	0.261	0.007	0.083	0.098	0.177
smms	$n = 1000$	10th	0.004	0.008	0.007	0.033	0.005	0.009	0.007	0.027
		50th	0.013	0.015	0.026	0.045	0.014	0.017	0.024	0.035
		90th	0.009	0.010	0.017	0.063	0.011	0.010	0.019	0.039
	$n = 2000$	10th	0.003	0.006	0.005	0.025	0.003	0.005	0.005	0.019
		50th	0.010	0.012	0.018	0.035	0.010	0.009	0.016	0.023
		90th	0.007	0.008	0.012	0.048	0.008	0.006	0.012	0.027
BayesTSM	$n = 1000$	10th	0.004	0.009	0.006	0.037	0.005	0.008	0.008	0.026
		50th	0.013	0.016	0.024	0.071	0.013	0.015	0.026	0.035
		90th	0.008	0.010	0.016	0.070	0.009	0.009	0.022	0.044
	$n = 2000$	10th	0.004	0.006	0.006	0.028	0.003	0.006	0.005	0.018
		50th	0.011	0.012	0.020	0.032	0.010	0.010	0.020	0.024
		90th	0.008	0.007	0.014	0.051	0.008	0.005	0.016	0.031
hmm	$n = 1000$	10th	0.004	0.018	0.009	0.044	0.004	0.098	0.006	0.047
		50th	0.013	0.053	0.034	0.286	0.015	0.051	0.024	0.222
		90th	0.010	0.015	0.022	0.601	0.011	0.028	0.021	0.442
	$n = 2000$	10th	0.003	0.016	0.005	0.035	0.003	0.010	0.004	0.042
		50th	0.009	0.053	0.018	0.291	0.010	0.050	0.019	0.219
		90th	0.006	0.010	0.012	0.607	0.008	0.028	0.016	0.437

Note: $p = 0$, no covariate; $p = 2$, two covariates.

3.9 Table showing the 95% coverage rates for the marginal percentiles of the cumulative incidence function for exponential and Weibull models under strong censoring when $p = 0$ and $p = 2$

Table S5. 95% coverage rates for the marginal percentiles of the cumulative incidence function for exponential and Weibull models under strong censoring when $p = 0$ and $p = 2$.

			$p = 0$				$p = 2$			
			state 1 → state 2		state 2 → state 3		state 1 → state 2		state 2 → state 3	
Method	Sample size	Percentile	Exponential	Weibull	Exponential	Weibull	Exponential	Weibull	Exponential	Weibull
msm[a]	$n = 1000$	10th	93	0	96	0				
		50th	93	73	97	5				
		90th	95	0	98	0				
	$n = 2000$	10th	90	0	95	0				
		50th	95	31	94	0				
		90th	94	0	93	0				
msm-phase[b]	$n = 1000$	10th								
		50th								
		90th								
	$n = 2000$	10th								
		50th								
		90th								
cthmm[a]	$n = 1000$	10th	61	0	72	0				
		50th	74	49	67	10				
		90th	58	0	50	0				
	$n = 2000$	10th	58	0	68	0				
		50th	69	18	68	1				
		90th	66	0	52	0				
smms[b]	$n = 1000$	10th								
		50th								
		90th								
	$n = 2000$	10th								
		50th								
		90th								
BayesTSM	$n = 1000$	10th	100	100	99	92	100	98	100	91
		50th	100	100	98	95	100	95	97	94
		90th	100	98	98	91	100	93	99	92
	$n = 2000$	10th	100	100	99	96	100	93	100	96
		50th	99	98	93	92	99	89	98	94
		90th	99	98	94	92	98	91	98	92
hmm	$n = 1000$	10th	95	81	93	93	87	0	90	87
		50th	94	5	91	24	95	0	91	17
		90th	87	79	92	0	94	24	92	1
	$n = 2000$	10th	91	76	98	93	92	0	91	91
		50th	97	1	99	3	93	0	89	0
		90th	97	77	98	0	96	24	88	0

Note: $p = 0$, no covariate; $p = 2$, two covariates.

[a] The 95% coverage rate (CR) is the proportion of the 95% confidence intervals (CIs) for the marginal percentiles of the CIF that contain the true percentile of the marginal CIF across the 100 simulated datasets. However, for models with covariates, there is no function available to directly compute this estimate.

[b] There is no function in this package to directly compute the asymptotic standard error of the CIF, which is needed for computing the 95% CI; hence the empty cells in the table.

3.10 Table showing the 95% coverage rates for the marginal percentiles of the cumulative incidence function for exponential and Weibull models under medium censoring when $p = 0$ and $p = 2$

Table S6. 95% coverage rates for the marginal percentiles of the cumulative incidence function for exponential and Weibull models under medium censoring when $p = 0$ and $p = 2$.

			$p = 0$				$p = 2$			
			state 1 $\to$ state 2		state 2 $\to$ state 3		state 1 $\to$ state 2		state 2 $\to$ state 3	
**Method**	**Sample size**	**Percentile**	**Exponential**	**Weibull**	**Exponential**	**Weibull**	**Exponential**	**Weibull**	**Exponential**	**Weibull**
msm[a]	$n = 1000$	10th	93	0	96	0				
		50th	93	0	96	16				
		90th	95	0	95	0				
	$n = 2000$	10th	88	0	98	0				
		50th	92	0	97	0				
		90th	93	0	96	0				
msm-phase[b]	$n = 1000$	10th								
		50th								
		90th								
	$n = 2000$	10th								
		50th								
		90th								
cthmm[a]	$n = 1000$	10th	52	0	67	0				
		50th	64	0	67	18				
		90th	61	0	56	0				
	$n = 2000$	10th	49	0	72	0				
		50th	68	0	63	8				
		90th	73	0	56	0				
smms[b]	$n = 1000$	10th								
		50th								
		90th								
	$n = 2000$	10th								
		50th								
		90th								
BayesTSM	$n = 1000$	10th	100	99	100	94	100	94	100	95
		50th	100	99	98	93	100	85	97	92
		90th	100	100	99	95	100	94	95	91
	$n = 2000$	10th	100	100	99	92	100	94	100	93
		50th	99	99	96	97	100	92	97	93
		90th	99	97	95	95	99	95	96	90
hmm	$n = 1000$	10th	94	51	93	91	82	0	95	49
		50th	94	13	94	7	89	3	97	6
		90th	95	83	93	0	86	25	96	0
	$n = 2000$	10th	90	24	96	80	83	0	93	19
		50th	96	0	96	0	91	0	93	0
		90th	95	85	95	0	89	10	95	0

Note: $p = 0$, no covariate; $p = 2$, two covariates.

[a] The 95% coverage rate (CR) is the proportion of the 95% confidence intervals (CIs) for the marginal percentiles of the CIF that contain the true percentile of the marginal CIF across the 100 simulated datasets. However, for models with covariates, there is no function available to directly compute this estimate.

[b] There is no function in this package to directly compute the asymptotic standard error of the CIF, which is needed for computing the 95% CI; hence the empty cells in the table.

4 Additional simulation study

As requested by a reviewer, we conducted an additional simulation study to (1) further compare the performance of the `smms` and `BayesTSM` packages, and (2) compare the cumulative hazard functions across all methods.

4.1 `smms` vs. `BayesTSM`

Results from the simulation study presented in the main manuscript showed that all methods performed well overall across settings assuming Markov models (i.e., exponential distributions) for the progression times (x, t). However, when semi-Markov models (i.e., Weibull distributions) were assumed, only `smms` and `BayesTSM` demonstrated good performance. In contrast, in the real cancer data application, only `BayesTSM` yielded converged solutions, whereas `smms` showed numerical and convergence issues. To better understand the difference in performance between `smms` and `BayesTSM`, we conducted an additional simulation study using settings that closely mirror the real-data example. Specifically, we assumed $p = 2$, Weibull-distributed progression times (x, t), and a sample size of $n = 734$. The parameters $(\theta, c_{\min}, c_{\max})$, as previously defined, were set to $(21.5, 1, 4.6)$, which resulted in approximately 65% of individuals in state 1, 28% in state 2, and 7% in state 3.

Similar to the simulation setup described in Section 2 above and in the main manuscript, this additional simulation study used 100 simulated datasets, each with a sample size of $n = 734$, three randomly initialized starting values for `smms`, and the same MCMC settings for `BayesTSM`. The `smms` package successfully converged to the global optima in 38 out of the 100 datasets; however, when the sample size was doubled to $n = 1468$, convergence improved to 59 out of the 100 datasets. For `BayesTSM`, each of the three MCMC chains was initially run with 10^5 iterations, with a maximum cap of 2×10^6 iterations per chain. Convergence was achieved in 95 out of the same 100 datasets; this increased slightly to 96 out of the 100 datasets when the sample size was doubled to $n = 1468$.

These results support our hypothesis that the convergence problems encountered with `smms` during model estimation in the real data application were likely due to the smaller sample size.

4.2 Cumulative hazard functions

In the main manuscript, we compared the various methods using the cumulative incidence function (CIF) across different simulation settings. In this section, we present results (see Figures S7 to S10) for the cumulative hazard functions under the exact same simulation settings.

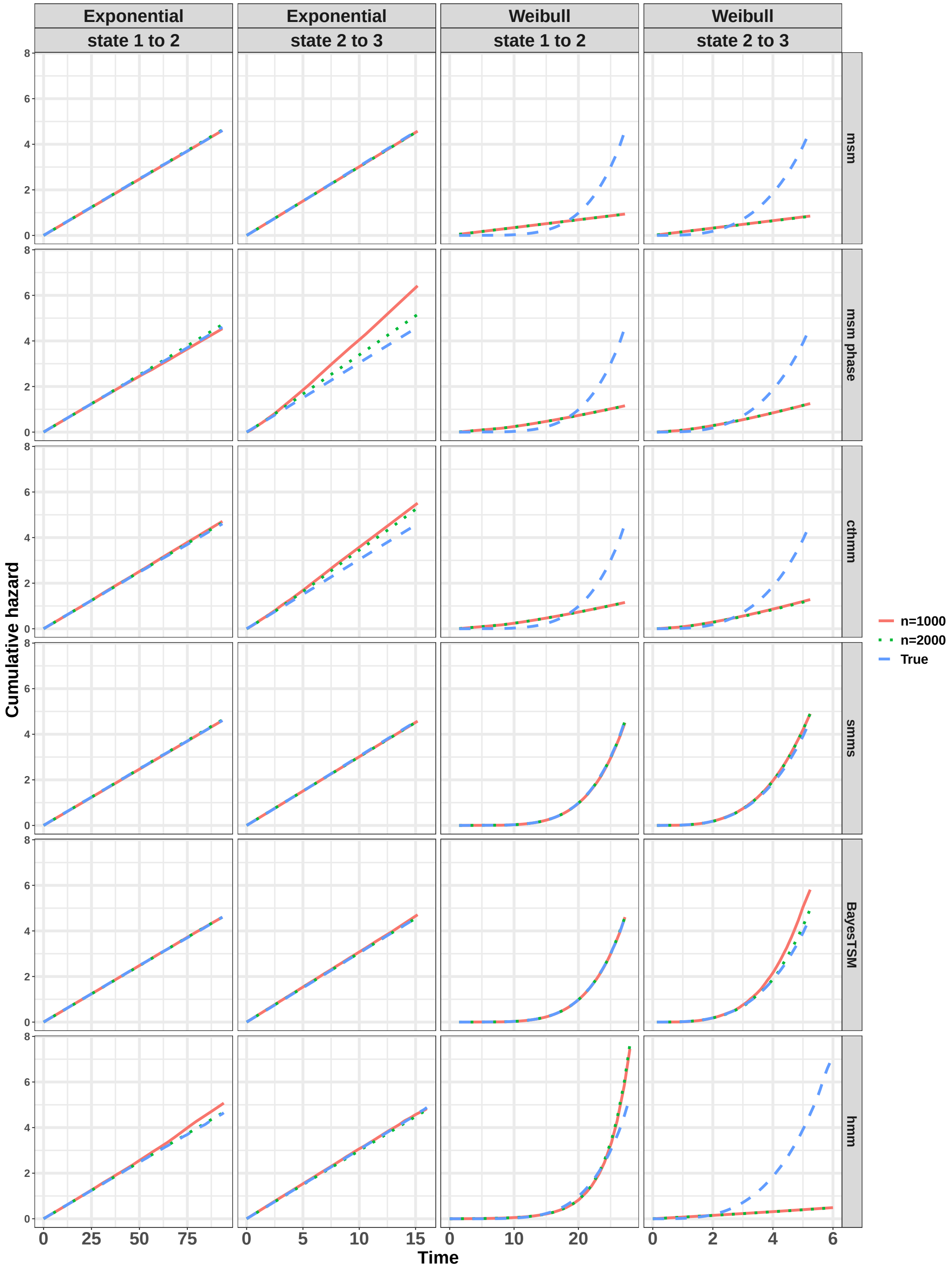

Figure S7. Marginal cumulative hazard functions compared for all six methods under strong censoring when $p = 0$.

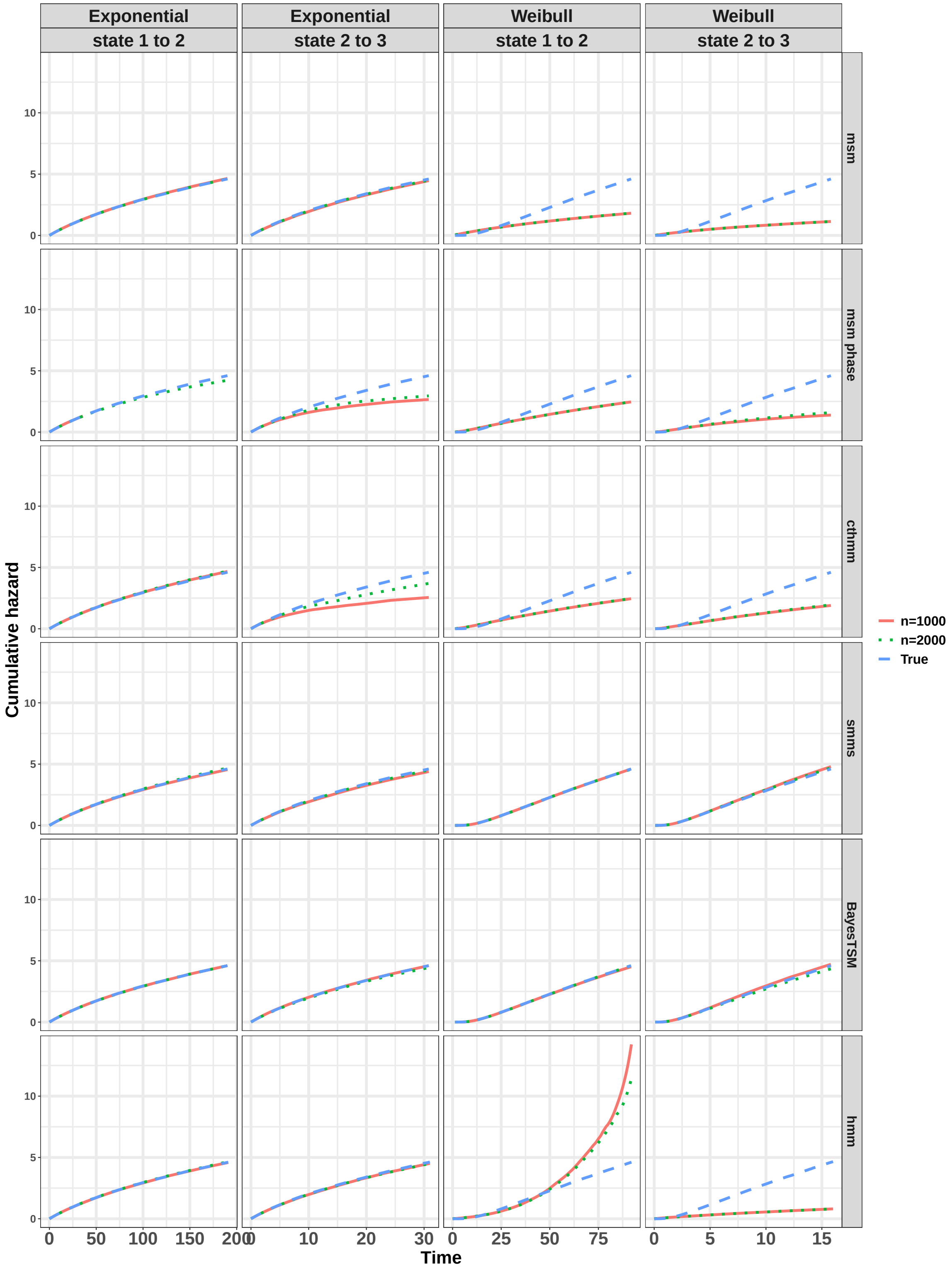

Figure S8. Marginal cumulative hazard functions compared for all six methods under strong censoring when $p = 2$.

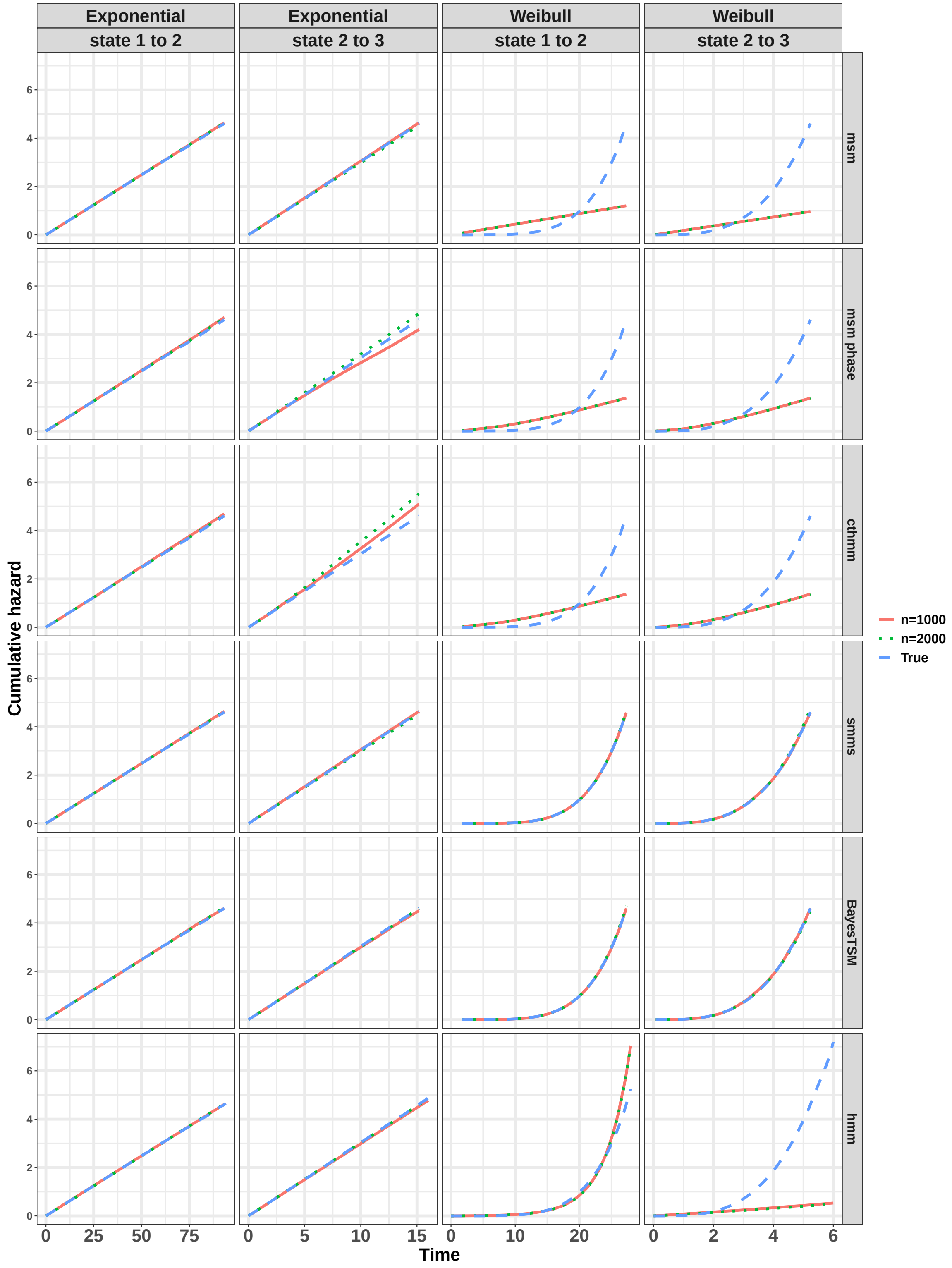

Figure S9. Marginal cumulative hazard functions compared for all six methods under medium censoring when $p = 0$.

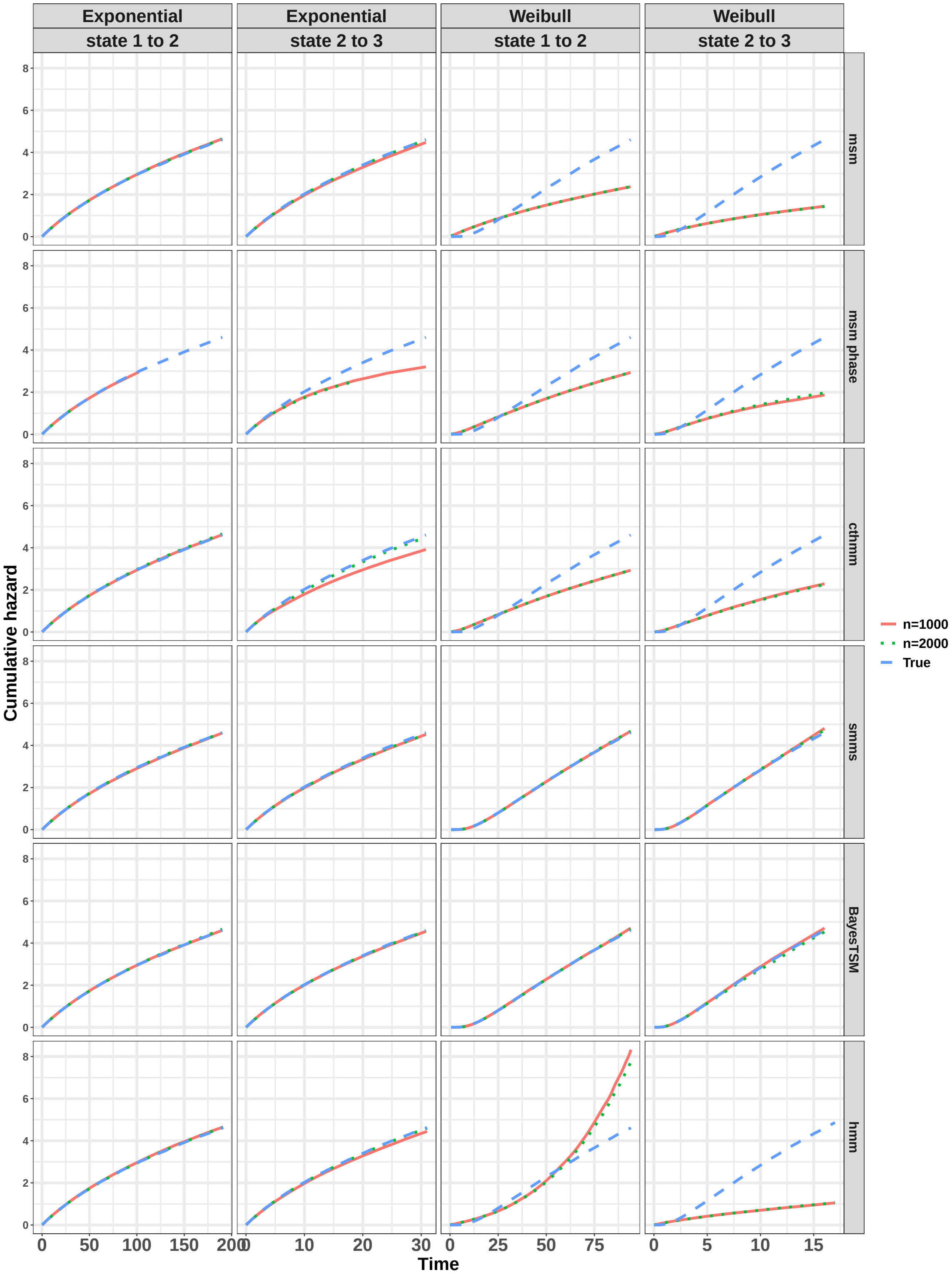

Figure S10. Marginal cumulative hazard functions compared for all six methods under medium censoring when $p = 2$.

5 An application to colorectal cancer progression

5.1 Characteristics of the 734 individuals included in the analysis

Table S7 presents the characteristics of the 734 individuals included in the analysis (Section 4 of the main manuscript) at their first colonoscopy, categorized according to the finding at the time of censoring during surveillance. These individuals are recommended to undergo direct surveillance with colonoscopy rather than attending routine screening, as they are at increased risk of developing adenomas or CRC compared to the general population (7). The 734 individuals in the dataset were pooled from the Dutch Familial Colorectal Cancer Surveillance (FACTS) randomized controlled trial ($n = 550$) (8) and the observational data collected at the Dutch Radboudumc and Rijnstate hospitals ($n = 286$).

Table S7. Characteristics of individuals at their first colonoscopy according to the finding at the time of censoring.

	Finding at time of censoring			
**Characteristics**	**Healthy** (n=477)	**nAA** (n=208)	**AN** (n=49)	**Total** (n=734)
% of total	65.0	28.3	6.7	100
Birth year, median (IQR)	1952 (1948 – 1957)	1950 (1946 – 1955)	1951 (1948 – 1956)	1951 (1947 – 1956)
Age, yr, mean (SD)	52.6 (7.2)	53.7 (7.9)	54.6 (6.9)	53.0 (7.4)
Sex, n (%)				
Male	206 (43.2)	92 (44.2)	29 (59.2)	327
Female	271 (56.8)	116 (55.8)	20 (40.8)	407
Findings at first colonoscopy, n (%)				
AA	16 (3.4)	8 (3.8)	9 (18.4)	33
nAA	54 (11.3)	66 (31.7)	9 (18.4)	129
No	407 (85.3)	134 (64.4)	31 (63.3)	572

AA: advanced adenoma; **AN:** advanced neoplasia; **nAA:** non-advanced adenoma; **IQR:** interquartile range; **SD:** standard deviation.

5.2 Results of the conditional cumulative incidence function using BayesTSM package

As an example, we computed the conditional CIFs for male individuals born in different years (1947, 1951, 1956), which represent the 25th, 50th, and 75th percentiles of the birth year distribution, respectively. Figure S11 depicts the estimated conditional CIFs from `BayesTSM` for male individuals born in 1947, 1951, and 1956. We observe that the risk estimates increase as the birth year becomes more recent; however, this difference in risk estimates is statistically significant only for the transition from HE to nAA (Table 4 in the main manuscript). For example, for male individuals born in 1947, the estimated risk of developing nAA up until the age of 55 was 23.5% (95% CrI: 19.3% – 28.3%). The corresponding risk estimates for male individuals aged 55 but born in later years were 29.3% (95% CrI: 25.3% – 33.9%) for those born in 1951, and 38.3% (95% CrI: 33.0% – 43.6%) for those born in 1956. One possible explanation for this increase is that individuals born earlier (e.g., 1947) may have undergone interventions or treatments in the past, prior to entering surveillance, which could have lowered their risk of developing nAA. Another explanation is the cohort effect, where the younger cohort may have experienced greater exposure to lifestyle factors such as smoking and less healthy diet, all of which are associated with an increased risk of colorectal cancer compared to the older cohort.

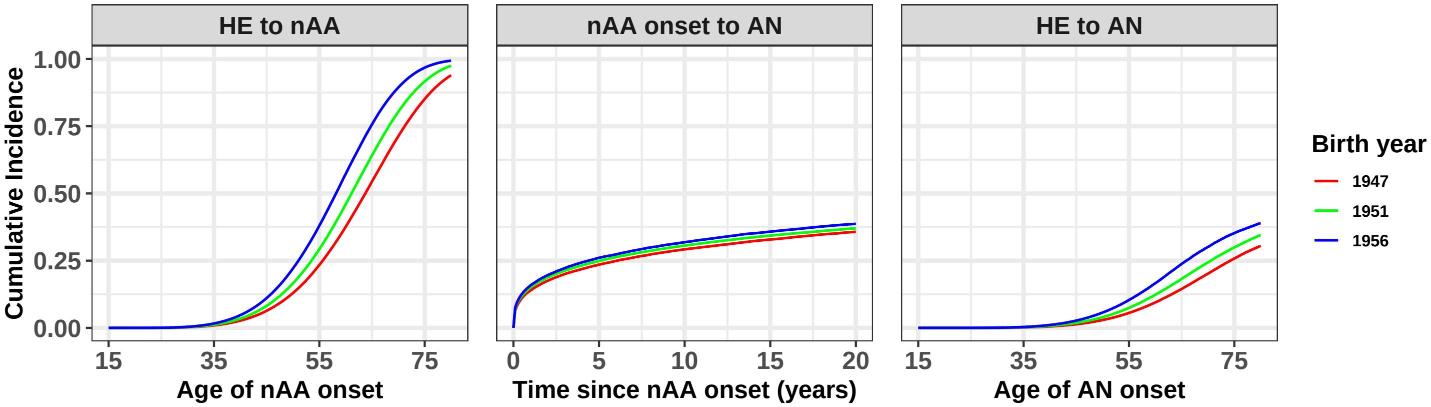

Figure S11. Estimated conditional cumulative incidence functions representing the personalized risks for male individuals born in different years (i.e., 1947, 1951 and 1956, correspond to the 25th, 50th, and 75th percentiles of the birth year distribution, respectively). The estimates were obtained using `BayesTSM`, based on a model that includes sex and (standardized) birth year as variables.

References

1. Klausch T, Akwiwu EU, van de Wiel MA, et al. A Bayesian accelerated failure time model for interval censored three-state screening outcomes. *Ann Appl Stat.* 2023;17(2):1285–1306.

2. Akwiwu EU, Klausch T, Jodal HC, et al. A progressive three-state model to estimate time to cancer: a likelihood-based approach. *BMC Med Res Methodol.* 2022;22(1):1–16.

3. Chen C, Yen M, Wang W, et al. A case–cohort study for the disease natural history of adenoma–carcinoma and *de novo* carcinoma and surveillance of colon and rectum after polypectomy: implication for efficacy of colonoscopy. *Br J Cancer.* 2003;88(12):1866–1873.

4. Nevala A, Heinävaara SH, Sarkeala T, et al. Bayesian hidden Markov model for natural history of colorectal cancer: handling misclassified observations, varying observation schemes and unobserved data. *Ann Appl Stat.* 2024;.

5. Collett D. *Modelling survival data in medical research.* CRC press; 2015.

6. Gelman A, Carlin JB, Stern HS, et al. *Bayesian data analysis.* Chapman and Hall/CRC; 1995.

7. van Leerdam ME, Roos VH, van Hooft JE, et al. Endoscopic management of Lynch syndrome and of familial risk of colorectal cancer: European Society of Gastrointestinal Endoscopy (ESGE) Guideline. *Endoscopy.* 2019;51(11):1082–1093.

8. Hennink SD, van der Meulen-de Jong AE, Wolterbeek R, et al. Randomized comparison of surveillance intervals in familial colorectal cancer. *J Clin Oncol.* 2015;33(35):4188–4193.
